# An eikonal model with re‐excitability for fast simulations in cardiac electrophysiology

**DOI:** 10.1113/JP287281

**Published:** 2026-09-01

**Authors:** Lia Gander, Rolf Krause, Francisco Sahli Costabal, Simone Pezzuto

**Affiliations:** ^1^ Euler Institute Università della Svizzera Italiana Lugano Switzerland; ^2^ AMCS, CEMSE King Abdullah University of Science and Technology Thuwal Saudi Arabia; ^3^ Department of Mechanical and Metallurgical Engineering Pontificia Universidad Católica de Chile Santiago Chile; ^4^ Department of Mathematics University of Trento Trento Italy; ^5^ Institute for Biological and Medical Engineering, Medicine and Biological Sciences Pontificia Universidad Católica de Chile Chile; ^6^ Millennium Institute for Intelligent Healthcare Engineering iHEALTH Chile

**Keywords:** anisotropic conduction, cardiac digital twins, cardiac electrophysiology, eikonal model, macro re‐entries, ventricular tachycardia

## Abstract

**Abstract:**

Precision cardiology based on cardiac digital twins relies on accurate yet computationally efficient simulations of cardiac electrophysiology and arrhythmias. Detailed biophysical models, such as the monodomain equation, are computationally demanding and therefore have limited applicability in clinical settings. It is thus desirable to develop reduced models that preserve the main physiological features of arrhythmic propagation while enabling faster computation. The standard eikonal model, an approximation of the monodomain model, effectively predicts the arrival times of the electrical wavefronts, but cannot reproduce the complex re‐entrant dynamics characteristic of many cardiac arrhythmias. In this work, we extend the eikonal model to include tissue re‐excitability, allowing it to capture re‐entries occurring during cardiac arrhythmias. Re‐excitability properties are inferred from the cable equation. The proposed formulation also incorporates tissue anisotropy and structural heterogeneities, such as scar and border zones. This structure separates the anatomical substrate for propagation from local restitution properties, which determine tissue recovery after excitation. When compared to the monodomain equation, our model reproduces anisotropic conduction and action potential duration with qualitative accuracy in cases involving macro re‐entries. Its computational efficiency and limited preprocessing requirements make it suitable for potential real‐time applications and integration within clinical digital twin frameworks.

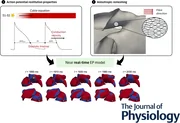

**Key points:**

- Re‐entry is a key mechanism sustaining cardiac arrhythmias, but detailed computer models can be too slow for patient‐specific clinical use.
- The eikonal model is fast, but normally describes only one activation wave and cannot reproduce repeated tissue excitation.
- We extend the eikonal model by adding tissue recovery, restitution properties and anisotropic conduction.
- The formulation separates local propagation, shaped by anatomy and conduction properties, from local tissue recovery, which can reflect cellular physiology.
- This fast approach reproduces major features of atrial and ventricular macro‐re‐entry and can support rapid screening of possible re‐entry circuits.

## Graphical abstract

**Abstract figure legend** Restitution properties obtained from cable‐equation simulations and anisotropic remeshing informed by local fibre orientation are combined in an eikonal model with re‐excitability. The resulting reduced electrophysiology model enables efficient simulations of recurrent activation patterns, here illustrated by atrial re‐entry snapshots.

## Introduction

Cardiac electrophysiology models are at the core of *in silico* patient‐specific clinical investigations, for example the understanding and the treatment of arrhythmias, such as atrial fibrillation (AF) (Boyle et al., 2019; Gharaviri et al., 2020, 2021; McDowell et al., 2015) or ventricular tachycardia (VT) (Ashikaga et al., 2013; Deng et al., 2019; Gionti et al., 2022; Trayanova et al., 2017). Patient‐specific computational models provide an instance of cardiac digital twins (CDTs) (Bhagirath, Strocchi, et al., 2024), a potentially transformative tool for clinical cardiology. A CDT utilizes our knowledge of physics and cardiac physiology while integrating multi‐modal clinical data, such as cardiac imaging and electrocardiography, into a predictive tool. CDTs could serve as the means to transition from population‐based cardiology to truly individualized care (Laubenbacher et al., 2024).

Despite their promise, CDTs remain elusive in clinical routine. One reason is that a CDT remains too computationally expensive to fit the strict clinical time frame. The most common model for cardiac arrhythmias is the monodomain equation, a complex system of ordinary and partial differential equations for the transmembrane potential and ionic model dynamics. However, the accurate biophysical detail enabled by the monodomain model demands a significant computational cost, mostly due to strict grid requirements (Pezzuto et al., 2016) to avoid unphysiological numerical artifacts (Bishop & Plank, 2025). This often hinders the applicability of the monodomain model to personalized clinical applications, where the results need to be delivered within tight time constraints.

There are alternatives that are less accurate but faster to speed up the computations. These can be approximate models based on simplified physics, such as the reaction‐eikonal model (Nagel et al., 2023; Neic et al., 2017) or the eikonal model (Nagel et al., 2023; Wallman et al., 2012), or simply models based on a coarser discretization of the computational domain in the numerical solution of the high‐fidelity equations (Gander et al., 2022; Pagani et al., 2021). An interesting approach is also the use of emulators (Álvarez‐Barrientos et al., 2025; Regazzoni et al., 2024; Salvador et al., 2024). Emulators serve as inexpensive approximations of high‐fidelity models, trained on a limited number of their samples. Since they are computationally efficient, emulators can dramatically accelerate the inference while maintaining sufficient accuracy. Unfortunately, an emulator trained for one patient cannot be directly reused for new patients because the anatomy varies between individuals. While it is theoretically possible to design emulators that include anatomy as a parameter (Banduc et al., 2025; Baranwal et al., 2024; Yin et al., 2024), doing so requires a large training dataset (potentially thousands of high‐fidelity simulations). This challenge becomes even more daunting when considering the need to account for combinations with other variable parameters. Additionally, emulators are typically tailored to specific quantities of interest and cannot fully capture the full predictive power of the original high‐fidelity model.

In this work, we start from the well‐established eikonal model for the activation times and extend it to account for repolarization and re‐entrant activity. The underlying eikonal equation is only space‐dependent, has no diffusion term and does not describe the ionic properties. Therefore, the numerical solution of the eikonal equation is cheaper than the numerical solution of the monodomain system (Neic et al., 2017). Our goal is to develop an eikonal model for cardiac arrhythmias and assess its quality by comparing it to the monodomain model.

Cardiac arrhythmias are characterized by a self‐sustained re‐entrant electrical activity, implying several consecutive tissue activations. The *standard* eikonal model describes a single wavefront activation, and therefore must be adapted for tissue re‐excitability and recurrent activations. The eikonal equation is typically solved by iterative algorithms that traverse the computational mesh, updating the activation times until acceptance. These methods are particularly suited for incorporating our proposed adaptations. To include the re‐excitability, a suitable iterative algorithm has to be single‐pass and has to guarantee the monotone acceptance of the nodes with respect to their activation times. The fast marching method (FMM) (Kimmel & Sethian, 1998; Sethian & Vladimirsky, 2000) satisfies these requirements and is therefore our method of choice.

From a numerical point of view, our adaptation of the FMM defines a global time variable and a time‐stepping that allows alternating between activation‐time updates and updates of the restitution properties, which describe the tissue re‐excitability. Our restitution properties are computed from the monodomain model. Moreover, our FMM approach handles the tissue anisotropy with mesh adaptation. Specifically, we use anisotropic meshes to guarantee the convergence of the FMM to the viscosity solution of the eikonal equation (Mirebeau, 2014). Additionally, the FMM also handles the tissue heterogeneity, such as due to structural remodelling. From a physiological perspective, this construction decouples two determinants of re‐entry that are often intertwined in full reaction–diffusion simulations: the local propagation pathway, governed by anatomy, conduction velocity, anisotropy and non‐conductive barriers, and the local recovery properties, governed by restitution of action potential duration and conduction velocity. This separation makes it possible to test how changes in tissue recovery, for instance those induced by electrophysiological remodelling or pharmacological action, alter the vulnerability of a given anatomical substrate to macro‐re‐entry.

Existing works in this direction have also proposed fast simulations of re‐entries by combining the monodomain and the eikonal models. In Jacquemet (2010) and Jacquemet (2012), the monodomain model is combined with the eikonal‐diffusion model to generate re‐entries along prescribed pathways. In Corrado & Zemzemi (2018), a reaction‐eikonal model is used to simulate a train of stimuli. The same reaction‐eikonal model is used in Gassa et al. (2021) to simulate spiral waves. In Barrios Espinosa et al. (2022), the monodomain and the eikonal models are alternated to generate a re‐entry on a ring. Recently, cellular automata have also been used to perform fast simulations of spiral waves (Romitti et al., 2023) and VT (Serra et al., 2022). Finally, the fast iterative method, commonly used in solving the standard eikonal equation, has been extended to account for re‐excitability (Barrios Espinosa et al., 2025).

Few works use a pure eikonal approach to carry out fast simulations with re‐excitability evaluations. In Corrado & Zemzemi (2018), an adapted Dijkstra method is used to simulate a train of stimuli. The FMM is adapted to simulate a train of stimuli in Sermesant et al. (2007), to initiate VT in Pernod et al. (2011) and to generate atrial flutter (AFlut) along precomputed re‐entry pathways in Loewe et al. (2019). In the present work, we use the FMM algorithm with re‐excitability to simulate various re‐entries and we propose a novel formal treatment of the anisotropy.

The rest of the paper is organized as follows. In the Methods section, we introduce the monodomain and the eikonal models, we compute the restitution curves of the monodomain model, and we present the FMM and its adaptation to include the re‐excitability. In the Numerical experiments section, we present some numerical experiments, in particular the simulations of VT and atrial flutter. A discussion follows in the Discussion section.

## Methods

### The monodomain equation

We start by introducing our reference model for cardiac electrophysiology, namely the monodomain equation (Colli Franzone et al., 2014). Consider a domain $\Omega \subset R^{3}$, representing the active myocardium, and a time interval [0,T]. The monodomain equation for the transmembrane potential v:Ω×[0,T)→R reads as follows

(1) $$\left\{\begin{array}{c} \nabla \cdot \;\left(D_{m}\nabla v\right)=\beta \left(C_{m}\frac{\partial v}{\partial t}+I_{\mathrm{ion}}\left(v,w\right)-I_{\mathrm{app}}\right)\\ \;\mathrm{for}\;x\in \Omega ,\;0<t<T,\\ \;\frac{\partial w}{\partial t}=G\left(v,w\right),\;\mathrm{for}\;x\in \Omega ,\;0<t<T,\\ D_{m}\nabla v\cdot n=0,\;\mathrm{for}\;x\in \partial \Omega ,\;0<t<T,\\ v\left(x,0\right)=v_{0}\;\left(x\right),\;x\in \Omega ,\\ w\left(x,0\right)=w_{0}\;\left(x\right),\;x\in \Omega , \end{array}\right.$$

where $D_{m}:\Omega \to R^{3\times 3}$ is the monodomain conductivity tensor, symmetric positive‐definite, $C_{m}=1\;\mu F/cm^{2}$ is the membrane capacitance, $I_{\mathrm{app}}\left(x,t\right)$ is an applied transmembrane current per unit area and β=800/cm is the surface‐to‐volume ratio (Gharaviri et al., 2020). The non‐linear term $I_{\mathrm{ion}}\left(v,w\right)$ is the ionic transmembrane current per unit area, and it depends on the type of tissue under consideration. The ionic current is determined by the transmembrane potential v and a possibly large set of auxiliary variables w(x,t), which describe the gating of ion channels, intracellular calcium dynamics and other ionic concentration variables. We define the monodomain conductivity tensor as transversely isotropic as follows:

$$D_{m}\left(x\right)=\sigma_{m}^{t}\;\left(x\right)I+\left(\sigma_{m}^{l}\left(x\right)-\sigma_{m}^{t}\left(x\right)\right)f_{l}\left(x\right)f_{l}{\left(x\right)}^{⊺},$$

where $\sigma_{m}^{l,t}:\Omega \to R$ are respectively the longitudinal and transversal conductivities defined as:

$$\;\sigma_{m}^{t}=\frac{\sigma_{m}^{t,i}\sigma_{m}^{t,e}}{\sigma_{m}^{t,i}+\sigma_{m}^{t,e}}\;,\;\;\;\sigma_{m}^{l}=\frac{\sigma_{m}^{l,i}\sigma_{m}^{l,e}}{\sigma_{m}^{l,i}+\sigma_{m}^{l,e}}\;,$$

and $f_{l}:\Omega \to R^{3}$ is the fibre orientation. The superscript ${\left(\cdot \right)}^{i,e}$ refers to the intracellular and extracellular conductivities respectively.

### Restitution curves

Cardiac arrhythmias are characterized by a re‐entrant electrical activity that implies several activations. These activations are allowed by the re‐excitability of the tissue. In this work, we include the restitution properties describing the re‐excitability in the eikonal model, computed from the monodomain model. In particular, we compute the restitution curves that determine the action potential duration (APD) and the conduction velocity (CV) from the diastolic interval (DI).

The procedure to compute the restitution curves consists of a pre‐pacing at a basic cycle length (BCL) and a pacing protocol to determine the DI, APD and CV associated with various cycle lengths (CLs) (Cherry et al., 2008; Wilhelms et al., 2013; Xie et al., 2002). Specifically, the protocol consists of a sequence of stimuli at BCL, followed by one stimulus at the considered CL, which is progressively decreased. The APD is defined as the time interval during which the transmembrane potential v is above the −62 mV threshold. We compute the CV and APD restitution curves with a time step Δt=0.02ms in a 1D tissue strand of length 4 cm and discretized with spatial resolution h=0.05mm. The mesh size is sufficiently fine to minimize the error in CV (Pezzuto et al., 2016). The stimuli have size 0.1 cm and are delivered from one end of the strand. The CV is computed from the activation times at the centre of the two halves of the domain. The activation time is defined as the time when v reaches −62 mV. We use this S1–S2 type protocol because the proposed model is intended to describe the acute response of tissue to a premature activation during re‐entry. Dynamic restitution protocols, in contrast, characterize the response after sustained pacing at a fixed cycle length and may include slower memory effects, such as calcium and ionic concentration adaptation. For the short re‐entry simulations considered here, the relevant physiological quantity is therefore the local relation between the current DI and the APD and CV of the next activation.

In this work we consider three ionic models for three different tissue types. For the atrial tissue, we consider the Courtemanche–Nattel–Ramirez ionic model of the human atria (Courtemanche et al., 1998) adapted to describe the electric remodelling due to AF (Gharaviri et al. 2020) and to reduce numerical instabilities (Potse, 2019; Potse et al., 2019). For the ventricular tissue we consider two versions of the Mitchell–Schaeffer ionic model (Mitchell & Schaeffer, 2003), one for the healthy tissue and one for the border zone surrounding the non‐conductive scars that characterize the structurally remodelled tissue (Pernod et al., 2011).

In the computation of the Courtemanche restitution curves, the CL ranges between BCL=1s and $CL_{0}=0.1\;s$, and reduces with decrements of 10 ms. The number of pre‐pacing stimuli is 2000, whereas 20 stimuli are delivered at BCL before measuring the restitution properties at each CL. Moreover, the monodomain conductivity is $\sigma_{m}=1.5\;\mathrm{mS}/\mathrm{cm}$ (Gharaviri et al., 2020). In the computation of the Mitchell–Schaeffer restitution curves, the pre‐pacing is not needed, the CL ranges between BCL=1.5s and $CL_{0}=0.2\;s$, reducing with decrements of 10 ms, and the number of stimuli delivered at BCL before measuring the restitution properties at each CL is 5. The monodomain conductivities are set to achieve a CV of 50 cm/s in the healthy tissue and a CV of 30 cm/s in the border zone (Pernod et al., 2011) and are respectively $\sigma_{m}=0.46\;\mathrm{mS}/\mathrm{cm}$ and $\sigma_{m}=0.19\;\mathrm{mS}/\mathrm{cm}$. The restitution curves are shown in Fig. 1.

**Figure 1:**
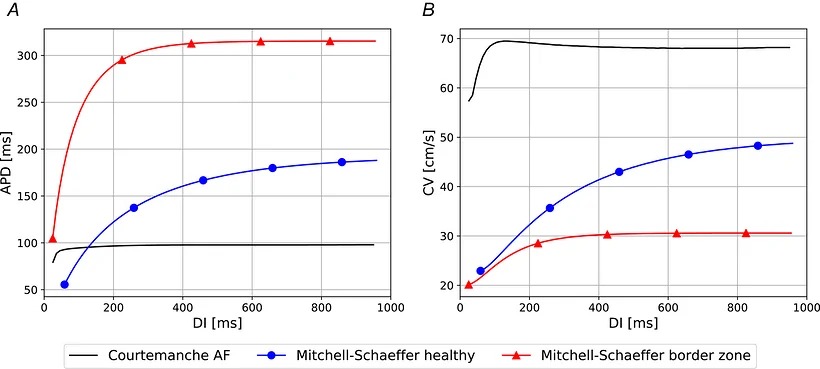
Restitution curves Restitution curves *A*, of the APD, and *B*, of the CV for the different ionic models considered.

The minimum DI for which the stimulation generates an action potential is $DI_{\min}=35\;\mathrm{ms}$ for the Courtemanche model, $DI_{\min}=60\;\mathrm{ms}$ for the Mitchell–Schaeffer model for the healthy tissue and $DI_{\min}=25\;\mathrm{ms}$ for the Mitchell–Schaeffer model for the border zone.

### Eikonal approximation

The eikonal approximation is given by the eikonal equation for the activation time ϕ:Ω→R, which reads (Colli Franzone et al., 2014)

(2) $$\left\{\begin{array}{cc} \;\sqrt{\nabla \phi^{⊺}D\nabla \phi }=1, & \mathrm{in}\;\Omega \setminus \Omega_{0},\\ \phi \left(\cdot \right)=\phi_{0}\;\left(\cdot \right), & \mathrm{in}\;\Omega_{0}, \end{array}\right.,$$

where $D:\Omega \to R^{3\times 3}$ is the anisotropy tensor given by

$$D\left(x\right)=CV_{t}^{2}\left(x\right)I+\left(CV_{l}^{2}\left(x\right)-CV_{t}^{2}\left(x\right)\right)f_{l}\left(x\right)f_{l}{\left(x\right)}^{⊺},$$

where $CV_{l,t}:\Omega \to R$ are respectively the longitudinal and transversal conduction velocities. The domain $\Omega_{0}\subset \Omega$ is the region where the action potential is initiated and $\phi_{0}:\Omega_{0}\to R$ is the initial value. Under suitable assumptions on the Dirichlet data, the eikonal equation has a unique viscosity solution, given by the Hopf–Lax formula (Bornemann & Rasch, 2006; Lions, 1982; Mirebeau, 2014)

$$\phi \left(x\right)=\underset{\begin{array}{c} \psi \in C^{1}\left(\left[0,1\right],\Omega \right),\\ \psi \left(0\right)\in \Omega_{0},\psi \left(1\right)=x \end{array}}{\inf}\;\mathrm{length}\left(\psi \right),$$

where the length of the path ψ connecting $\Omega_{0}$ to $x\in \Omega \setminus \Omega_{0}$ is

$$\mathrm{length}\left(\psi \right)=\int_{0}^{1}\sqrt{\psi^{\prime }{\left(s\right)}^{⊺}D^{-1}\left(\psi \left(s\right)\right)\psi^{\prime }\left(s\right)}\;\;ds.$$

### Fast marching method

The eikonal eqn (2) is typically solved by algorithms that iteratively pass through the nodes of a mesh until acceptance of the activation times. Our goal is to adapt an existing algorithm to account for the re‐excitability. A suitable algorithm has to be single‐pass and has to guarantee the monotone acceptance of the nodes with respect to their activation times. The FMM (Kimmel & Sethian, 1998; Sethian & Vladimirsky, 2000) is our choice, since it satisfies these requirements, contrary to the fast iterative method (FIM) (Fu et al., 2011) and the method presented in Bornemann & Rasch (2006) based on an adaptive Gauss–Seidel iteration. The FMM is a Dijkstra‐like method that considers updates coming from within the elements of the mesh, contrary to the standard Dijkstra method (Dijkstra, 1959) that only considers updates from the edges.

The pseudo‐code of the FMM is reported in Algorithm (1). The set of nodes is denoted by X and the set of nodes in $\Omega_{0}$ is denoted by $X_{0}\subset X$. Moreover, for a node x, N(x) denotes the set of neighbours of x, in particular $N(X_{0})$ are the neighbours of the initial points, and T(x) denotes the set of elements that have x as vertex. The anisotropy tensor D is assigned to the elements, whereas the nodes are assigned an activation time ϕ and to a state (*Far*, *Considered* or *Accepted*). The *Far* nodes have no activation time, the *Considered* nodes have a temporary activation time $\tilde{\phi }$ that has not yet been accepted and the *Accepted* nodes have a known activation time. At each iteration, the *Considered* node with minimum temporary activation time is marked as *Accepted*, its non‐*Accepted* neighbours are marked as *Considered* and their temporary activation time is updated with the Hopf–Lax formula, denoted by HL(ϕ,D).

**Table:** 

Algorithm 1: Fast marching method
*# Initialization*
Initialize $x\in X\setminus X_{0}$ as *Far* with ϕ(x)=∞
Initialize $x_{0}\in X_{0}$ as *Accepted* with $\phi \left(x_{0}\right)=\phi_{0}\;\left(x_{0}\right)$
*# Update the neighbours*
Tag $x_{n}\in N\left(X_{0}\right)\setminus \mathrm{Accepted}$ as *Considered*
Compute $\tilde{\phi }\left(x_{n}\right)=\;\mathrm{HL}\left(\phi \left(N(x_{n})\right),D\left(T(x_{n})\right)\right)$
*# Iterate over the nodes*
**While** Considered ≠∅ do
Tag $x_{a}=\mathrm{argmi}n_{x\in \mathrm{Considered}}\tilde{\phi }\left(x\right)$ as *Accepted* with $\phi \left(x_{a}\right)=\tilde{\phi }\;\left(x_{a}\right)$
Tag $x_{n}\in N\left(x_{a}\right)\setminus \mathrm{Accepted}$ as *Considered*
Compute $\tilde{\phi }\left(x_{n}\right)=\;\mathrm{HL}\left(\phi \left(N(x_{n})\right),D\left(T(x_{n})\right)\right)$

The temporary activation time is computed from a local discretization of the Hopf–Lax formula (Mirebeau, 2014)

(3) ϕ∼x=miny∈∂MxIMxϕy+(x−y)⊺D−1Tx,yx−y+(x−y)⊺D−1Tx,yx−y,

where M(x) is the mesh of a small neighbourhood of $x\in X\setminus X_{0}$, $I_{M(x)}$ is the piecewise linear interpolation on M(x) and T(x,y) denotes the element having x as vertex and y∈∂M(x) on its boundary. The actual solution of the local minimization problem in eqn (3) is carried out analytically (Grandits et al., 2021, 2024).

The convergence of the FMM to the viscosity solution of the eikonal eqn (2) is guaranteed if all the elements T of the mesh satisfy

(4) $$e_{i}^{⊺}D^{-1}\left(T\right)e_{j}>0$$

for all the edges $e_{i,j}$ of a common face of T. Inequality (4) is an acuteness condition with respect to the metric defined by $D^{-1}$. Our approach to deal with this condition is to use the anisotropic mesh adaptivity (Dobrzynski & Frey, 2008) to generate a mesh maximizing the number of elements that satisfy the acuteness condition with respect to the specific metrics of our applications.

To illustrate the effect of the mesh adaptation and of the updates coming from within the triangles, we consider the simple case of a propagation on a square with side length of 1 cm. The action potential is initiated on the lower left corner, the fibres are diagonally oriented, the longitudinal CV is $C\;V_{l}=65\;\mathrm{cm}/s$ and the anisotropy ratio is $CV_{t}/C\;V_{l}=0.33$. We compute the eikonal solution with the FMM on an adapted mesh, the FMM on a non‐adapted mesh and the edge Dijkstra's method on the adapted mesh. The corresponding activation times are shown in Fig. 2.

**Figure 2:**
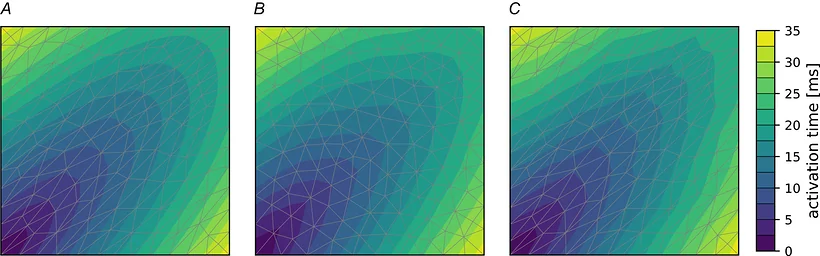
Single‐pass methods do not necessarily satisfy the causality condition, possibly yielding incorrect solutions *A*, contour plots of the activation times showing an example obtained with the FMM on an adapted mesh; *B*, the FMM on a non‐adapted mesh; and *C*, Dijkstra's method on the adapted mesh.

The solution obtained with the FMM on the adapted mesh is more accurate than the one computed on the non‐adapted mesh and the one obtained with Dijkstra's method.

### Fast marching with re‐excitability

Our adaptation of the FMM to include the re‐excitability requires several modifications to Algorithm (1). The re‐excitability is determined by the APD and DI, which are assigned to the nodes. When a node activates, the APD determines the time interval during which it is active and the DI determines when it becomes re‐excitable. The APD depends on the DI through the restitution curves shown in Fig. 1*A*, and denoted by the function a, which depends on the tissue type of the node, which we denote by TT. The minimum diastolic interval, $DI_{\min}$ determining the re‐excitability also depends on the tissue type. The activation time ϕ is computed at each beat. In particular, it takes a finite value when the nodes are active and the value ∞ when the nodes are excitable. The state *Far* is assigned to the excitable nodes, the state *Considered* is assigned to the excitable nodes that have non‐accepted a temporary activation time $\tilde{\phi }$ and the *Accepted* state is assigned to the non‐excitable nodes. To facilitate the comparison between the eikonal and monodomain models, the nodes are also assigned a pseudo‐potential v taking the value 1 when active and the value 0 when inactive. The anisotropy tensor D is again assigned to the elements. The longitudinal conduction velocity $CV_{l}$ depends on the DI through the restitution curves shown in Fig. 1 *B*, and denoted by the function c, which depends on the tissue type.

The temporary activation time of a *Considered* node x is computed when a neighbour $x^{\prime }$ activates. When computing the temporary activation time of x, the $CV_{l}$ of the elements having x as vertex need to be known. Since the $CV_{l}$ assigned to the elements depends on the DI assigned to the nodes, a single element might take different $CV_{l}$ values depending on which node is being considered. Therefore, we use the notation $T(x,x^{\prime })$ to denote the set of elements having both x and $x^{\prime }$ as vertices and to refer to the propagation from $x^{\prime }$ to x. The $CV_{l}$ assigned to the elements in $T(x,x^{\prime })$ depends on the DI of x at the moment when $x^{\prime }$ activates. Moreover, we use the notation T(x,:) to denote the set of elements having x as vertex and to refer to the propagation to x.

The adapted FMM needs a global time variable in order to determine when the action potential on an activated node is over, to compute the DI and to determine when a previously activated node becomes re‐excitable. These updates are not performed at each fast marching iteration, but rather a time‐stepping is used; that is, the updates are performed at the end of each time step. The pseudo‐code of the FMM with re‐excitability is reported in Algorithm (2). The algorithm makes use of the restitution curves c(DI) and a(DI), respectively. for the conduction velocity and the APD, at a given diastolic interval DI. The curves are linearly interpolated. The current time of the simulation is denoted by t, the time step is denoted by Δt and the final time of the simulation is denoted by T. The algorithm handles several stimuli. The number of stimuli is denoted by $N_{\mathrm{stim}}$. For $i=1,\text{…},N_{\mathrm{stim}}$, the ith stimulus is delivered at $\Omega_{i}\subset \Omega$ at time $t_{i}$. The set of nodes in $\Omega_{i}$ is denoted by $X_{i}\subset X$ for $i=1,\text{…},N_{\mathrm{stim}}$.

**Table:** 

Algorithm 2: Fast marching with re‐excitability
Initialize x∈X as *Far* with ϕ(x)=∞, v(x)=0 and DI(x)=∞.
Set $t=t_{1}$.
**While** t<T:
* # Check for stimuli*
**If** $t_{i}\in \left[t,t+\Delta t\right)$ for some $1\leq i\leq N_{\mathrm{stim}}$:
Tag $x_{i}\in X_{i}\setminus Accepted$ as *Accepted* with $\phi \;\left(x_{i}\right)\;=\;t_{i}$, $v\;(x_{i})\;=\;1$,
$APD\;\left(x_{i}\right)=a\left(TT\left(x_{i}\right),DI\left(x_{i}\right)\right)$, and $DI\;(x_{i})=0$.
Tag $x_{n}\in N\left(X_{i}\right)\setminus Accepted$ as *Considered*.
Set $CV_{l}\;\left(T(x_{n},x_{i})\right)=c\left(TT\left(x_{n}\right),DI\left(x_{n}\right)\right)$.
Compute $\tilde{\phi }\;\left(x_{n}\right)=\;\mathrm{HL}\left(\phi \left(N(x_{n})\right),D\left(T(x_{n})\right)\right)$.
* # Iterate within one time step*
**While** $\mathrm{mi}n_{x\in \mathrm{Considered}}\tilde{\phi }\left(x\right)<t+\Delta t$:
Tag $x_{a}=\mathrm{argmi}n_{x\in \mathrm{Considered}}\;\tilde{\phi }\left(x\right)$ as *Accepted* with $\phi \;\left(x_{a}\right)=\tilde{\phi }\;\left(x_{a}\right)$, $v\;(x_{a})=1$
$APD\;\left(x_{a}\right)=a\left(TT\left(x_{a}\right),DI\left(x_{a}\right)\right)$, $CV_{l}\;\left(T(x_{a},:)\right)=0$, and $DI\;(x_{a})=0$.
Tag $x_{n}\in N\left(x_{a}\right)\setminus Accepted$ as *Considered*.
Set $CV_{l}\;\left(T(x_{n},x_{a})\right)=c\left(TT\left(x_{n}\right),DI\left(x_{n}\right)\right)$.
Compute $\tilde{\phi }\;\left(x_{n}\right)=\;\mathrm{HL}\left(\phi \left(N(x_{n})\right),D\left(T(x_{n})\right)\right)$.
* # Updates for the next time step*
Set t=t+Δt.
For x∈Accepted such that ϕ(x)+APD(x)<t, set v(x)=0 and DI(x)=t−(ϕ(x)+APD(x)).
For x∉Accepted, set DI(x)=DI(x)+Δt.
Tag x∈Accepted such that $DI\left(x\right)\geq DI_{\min}\left(TT(x)\right)$ as *Far* with ϕ(x)=∞.

### Clinical data

We considered two patient‐specific models: a case of a left ventricle with scars in the septal region, and a left‐atrial model. Both are based on previously described clinical data.

The ventricular model is one case of a cohort of patients who underwent cardiac resynchronization therapy (Maffessanti et al., 2020; Pezzuto et al., 2021). The institutional review board approved the study protocol, and all patients gave written and oral informed consent for the investigation. The study was performed in compliance with the *Declaration of Helsinki*. The anatomical model (heart, torso, lungs, major blood masses) was reconstructed from cardiac magnetic resonance (CMR) scan with late gadolinium‐enhancement (LGE). Surfaces of relevant anatomical structures were constructed from their contours using Blender (The Blender Foundation). The outline of the ventricles was detected from short‐axis cine images. Scarred tissue and border zone were inferred from LGE magnetic resonance imaging (LGE‐MRI) sequence by manual contour and alignment. Ventricular fibre orientation was assigned using a rule‐based approach (Potse et al., 2014). The left‐ventricular surface model was based on the mid‐myocardium of the full 3‐D model. The valvular plane was closed to facilitate the re‐entry.

The atrial model is openly accessible on Zenodo (Martínez Díaz et al., 2024). Specifically, we selected case ‘P1’, only the left atrium. We modified the original bilayer by extracting only the endocardium. We used the Mmg software (Dobrzynski & Frey, 2008) to refine the original mesh, obtaining a grid with average edge size of 0.05 cm and approx. 93,000 nodes. The fibre, sheet and tag fields were interpolated from the original mesh onto the refined one with radial basis functions, as implemented in the software pyvista.

## Numerical experiments

We test the performance of the eikonal model with re‐excitability in several numerical experiments. We compare the eikonal results to the monodomain results in some simulations of re‐entrant electrical activity.

The monodomain implementation uses linear finite elements for the spatial discretization of the diffusion term of the monodomain equation and the implicit Euler method for the time integration of the transmembrane potential. The implicit time step consists in solving a parabolic equation, with full mass matrix and interpolated currents (Pezzuto et al., 2016). The corresponding linear system is solved with the conjugate gradient method and incomplete LU (ILU) preconditioner. The Courtemanche ionic model is implemented in the CUDA‐based Propag‐5 interface (Krause et al., 2012; Potse et al., 2006), where, for the time integration, the Rush–Larsen method (Rush & Larsen, 1978) is used for the gating variables and the explicit Euler method is used for the concentration variables. Overall, the discretization is first‐order accurate in time and second‐order in space. The Mitchell–Schaeffer ionic model is implemented in Python, using the explicit Euler method for the time integration of the gating variable.

The FMM with re‐excitability of Algorithm (2) is implemented in Python. The FMM implementation relies on a min‐heap structure to efficiently find the *Considered* node with minimal temporary activation time at each iteration. We use the heap class in the scikit‐fmm module, which allows the values in the heap to be updated.

In all the numerical experiments, the computational domain is discretized by a triangulated mesh with average edge length of h=0.05cm. The time step is Δt=0.02ms in the monodomain simulations and Δt=1ms in the eikonal simulations.

### Re‐entries in the presence of scars

We first test our model in the presence of re‐entrant activity around non‐conductive scars, which represent the dynamics underlying VT. Therefore, we consider computational domains with scars, border zone and healthy tissue. Compared to healthy tissue, the border zone is characterized by a longer APD and a lower CV. In the monodomain model, the ionic properties are described by two Mitchell–Schaeffer ionic models. In the eikonal model, the restitution properties are described by the corresponding restitution curves (see the restitution curves section). The longitudinal monodomain conductivities are $\sigma_{m,h}^{l}=0.46\;\mathrm{mS}/\mathrm{cm}$ in the healthy tissue and $\sigma_{m,\mathrm{bz}}^{l}=0.19\;\mathrm{mS}/\mathrm{cm}$ in the border zone. The ratio between the transversal and the longitudinal monodomain conductivities is $\sigma_{m}^{t}/\;\sigma_{m}^{l}=1/9$ in both the healthy tissue and the border zone (Pernod et al., 2011).

#### The 2‐D case

We first consider the 2‐D case of a square with side length of 15cm. The domain has two rectangular scars of size 3×5cm located in the middle, with a border zone of width 1cm between them, as illustrated in Fig. 3.

**Figure 3:**
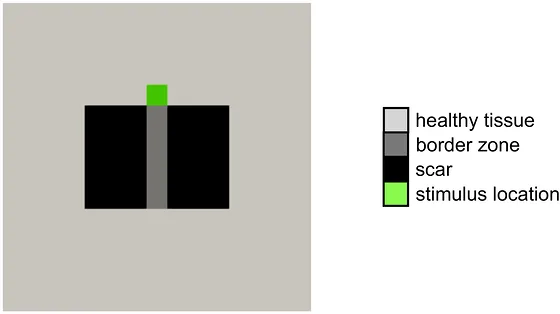
Geometrical setup for the simulation of a re‐entry circuit passing through the isthmus The square of side 15 cm has two scars, non‐conductive, a border zone located in the channel and fibres running horizontally. A series of two stimuli is delivered in the green region at time t=0 and t=290ms.

We generate a mesh with the Mmg software (Dobrzynski & Frey, 2008) and we set horizontally oriented fibres, that is, $f_{l}={\left[1,0\right]}^{⊺}$. To compute the anisotropy ratios $CV_{t}/CV_{l}$, we simulate a monodomain propagation on the healthy and the border zone tissues and we compute the CVs in the directions longitudinal and transversal to the fibres. In the healthy tissue, the longitudinal CV is 49cm/s and the transversal CV is 30cm/s, so the anisotropy ratio is $CV_{t}/C\;V_{l}=0.61$. In the border zone, the longitudinal CV is 30cm/s and the transversal CV is 19cm/s, so the anisotropy ratio is $CV_{t}/C\;V_{l}=0.63$.

The isotropic mesh that we use in the monodomain simulation is not appropriate for the eikonal simulation, as the anisotropy tensor D is not proportional to the identity I. Indeed, the percentage of triangles not satisfying the acuteness condition (4) with respect to the metric defined by $D^{-1}$ is 52.7%. Therefore, we use the Mmg software to generate an anisotropic mesh that we use in the eikonal simulation. The resulting mesh has only 0.4% of triangles that do not satisfy the acuteness condition. Note that the anisotropic mesh is comparable to the isotropic mesh in terms of number of nodes.

The re‐entry is initiated by two stimuli of size 1×1cm applied above the border zone, as shown in Fig. 3. The first stimulus is applied at time t=0ms and the second stimulus is applied at time t=290ms. We perform the monodomain and the eikonal simulations on a laptop with T=2000ms. The monodomain and the eikonal simulations are shown in Fig. 4. Five snapshots show the active tissue in red and the inactive tissue in blue. In the monodomain simulation, the tissue is active when the transmembrane potential is above the −62mV threshold. In the eikonal simulation, the tissue is active when the pseudo‐potential equals 1.

**Figure 4:**
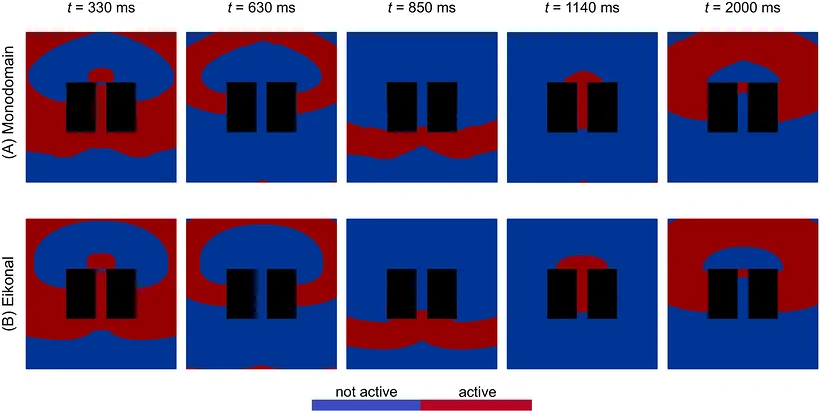
Re‐entry in the presence of scars *A*, monodomain and *B*, eikonal simulations of a re‐entry in the presence of scars.

When the second stimulus is applied, the border zone is still active, as its APD is longer than the APD of the healthy tissue. As a consequence, the action potential resulting from the second stimulus cannot initially propagate downwards. This dynamics is called unidirectional block. The time t=330ms is shortly after the second stimulus is applied. The action potential initially propagates only upwards and laterally, and subsequently rotates around the scars generating a re‐entry. The snapshots at times t=630ms, t=850ms and t=1140ms illustrate the rotation and the re‐entry that self‐sustains until the end of the simulation at time t=2000ms.

The action potential propagates as a convex front. The wavefront curvature affects the CV in the monodomain model. In particular, compared to a planar front, a convex front propagates with a reduced CV (Fast & Kléber, 1997). The eikonal model does not capture the curvature effects, as it does not account for the diffusion currents. Therefore, the action potential propagates slightly faster in the eikonal simulation than in the monodomain simulation. The difference between the monodomain and the eikonal shape of the depolarization and repolarization fronts is due to the effect of the space resolution on the monodomain simulation, see Appendix, Monodomain simulations.

#### Ventricular tachycardia

We now consider the case of the left ventricular surface with two scars and with border zones as illustrated in Fig. 5.

**Figure 5:**
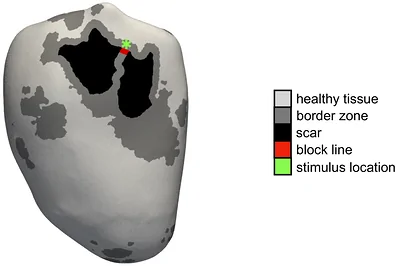
Ventricular tachycardia setup Left ventricle with two scars, border zones, block line and location of the stimulus.

We use the Mmg software (Dobrzynski & Frey, 2008) to refine the original coarse mesh, we map the tissue types from the coarse to the fine mesh and we set the circumferential fibre orientation as in the midwall. To compute the anisotropy ratios $CV_{t}/CV_{l}$ for the surface, we simulate two monodomain propagations on a square, similarly to the 2‐D case in the Re‐entries in the presence of scars section, but here with diagonally oriented fibres (see Appendix, Anisotropy ratios, for the motivation). In the healthy tissue, the longitudinal CV is 49cm/s and the transversal CV is 22cm/s, so the anisotropy ratio is $CV_{t}/C\;V_{l}=0.45$. In the border zone, the longitudinal CV is 30cm/s and the transversal CV is 14cm/s, so the anisotropy ratio is $CV_{t}/C\;V_{l}=0.47$. The anisotropy tensor D is again not proportional to the identity I. Therefore, the isotropic mesh that we use in the monodomain simulation is not suitable for the eikonal simulation, and indeed 64.0% of the triangles do not satisfy the acuteness condition. Instead, the anisotropic mesh generated with the Mmg software is suitable, as the percentage of triangles not satisfying the acuteness condition is only 3.4%. Thus, we map the tissue types from the isotropic to the anisotropic mesh and we assign the circumferential fibre orientation also to the anisotropic mesh.

We apply a single stimulation and we use the block line approach to simulate a unidirectional block that initiates VT. The block line and the location of the stimulus are shown in Fig. 5. The stimulus is applied at time t=0ms. The block line is non‐conductive at the beginning of the simulation and becomes conductive at time t=330ms. We perform the monodomain and the eikonal simulations on a laptop with T=2000ms. The monodomain and the eikonal simulations are shown in Fig. 6.

**Figure 6:**
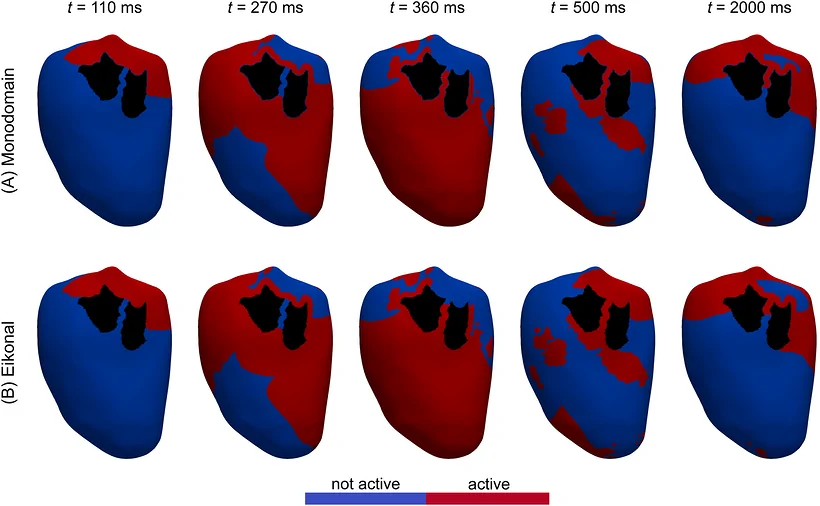
Ventricular tachycardia simulation *A*, monodomain and *B*, eikonal simulations of VT.

When the stimulus is applied, because of the block, the resulting action potential cannot propagate in the gap between the two scars. As a consequence, the action potential propagates around the scars, as illustrated by the snapshots at times t=110ms and t=270ms. The action potential reaches the location of the block line around time t=360ms, when the tissue is already conductive. Therefore, the action potential can continue the rotation around the scars, as shown in the snapshot at time t=500ms. This re‐entrant dynamics self‐sustains until the end of the simulation at time t=2000ms. Again, the eikonal action potential propagation is slightly faster than the monodomain propagation. This is again due to the fact that the wavefront is mostly convex and that the eikonal model does not capture the effect of the curvature.

### Spiral waves

The last numerical experiments are simulations of spiral waves, which characterize the dynamics of an atrial flutter (AFlut). Therefore, we consider computational domains of healthy atrial tissue. In the monodomain model, the ionic properties are described by the Courtemanche ionic model adapted to AF electric remodelling. In the eikonal model, the restitution properties are described by the corresponding restitution curves (see the restitution curves section). In the monodomain simulations, the initial conditions on v and w are obtained after the simulation of 2000 beats at BCL=1s in a single‐cell model. The longitudinal and transversal monodomain conductivities are respectively $\sigma_{m}^{l}=1.5\;\mathrm{mS}/\mathrm{cm}$ and $\sigma_{m}^{t}=0.24\;\mathrm{mS}/\mathrm{cm}$ (Gharaviri et al., 2020).

#### The 2‐D case

We first consider the 2‐D case of a square with side length of 15cm and horizontally oriented fibres, that is, $f_{l}={\left[1,0\right]}^{⊺}$. The isotropic mesh is the same as in  the 2‐D case above. We compute the anisotropy ratio $CV_{t}/CV_{l}$ with a monodomain simulation as described in the 2‐D case above. The longitudinal CV is 68cm/s, whereas the transversal CV is 34cm/s. Therefore, the anisotropy ratio is $CV_{t}/C\;V_{l}=0.5$. The isotropic mesh for the monodomain simulation has 74.8% of triangles that do not satisfy the acuteness condition with respect to the metric defined by $D^{-1}$, so it is not appropriate for the eikonal simulation. Instead the anisotropic mesh generated with the Mmg software (Dobrzynski & Frey, 2008) is appropriate, as the percentage of triangles not satisfying the acuteness condition is only 0.4%.

Similarly to Gassa et al. (2021), the spiral wave is initiated by a first stimulus with width 0.1cm on the left side of the domain at time t=0ms, followed by a second stimulus on the lower left rectangle of size 6×12cm at time t=210ms. We perform the monodomain and the eikonal simulations on a laptop with T=1000ms.

The monodomain and the eikonal simulations are shown in Fig. 7. Time t=210ms is the instant when the second stimulus is applied. Note that part of the stimulated tissue might not be excitable. In the monodomain case, the stimulation in the non‐excitable tissue results in a quick increase of the transmembrane potential, which then rapidly decreases back to its pre‐stimulation value, without affecting the overall dynamics. In the eikonal case, because of the implementation of Algorithm (2), the non‐excitable tissue is not activated. The action potential resulting from the second stimulus initially propagates only upwards and subsequently generates a re‐entry. At time t=305ms, the tissue where the second stimulus was applied is inactive, but it is not yet excitable, so the action potential only propagates towards the right and downwards. At time t=365ms, the stimulated tissue is excitable and the action potential propagates also towards the left. The re‐entry, generated around time t=425ms, induces a spiral wave that self‐sustains until the end of the simulation at time t=1000ms. The action potential resulting from the first stimulus propagates as a planar front, and therefore the eikonal model is accurate in describing the related depolarization and repolarization. Instead, in the spiral wave resulting from the second stimulus there are differences between the simulations that are due to the effect of the wavefront curvature on the monodomain CV, which is not captured by the eikonal model. On the one hand, since the action potential propagates as a convex front, the eikonal propagation is faster than the monodomain propagation (Fast & Kléber, 1997). On the other hand, the monodomain rotation is faster than the eikonal rotation, as the tip of the re‐entry moves in the monodomain simulation, whereas it is fixed in the eikonal simulation (Panfilov & Dierckx, 2018). Again, the shape of the depolarization and repolarization fronts is different in the monodomain and the eikonal cases because of the effect of the space resolution on the monodomain simulation (see Appendix, Monodomain simulations).

**Figure 7:**
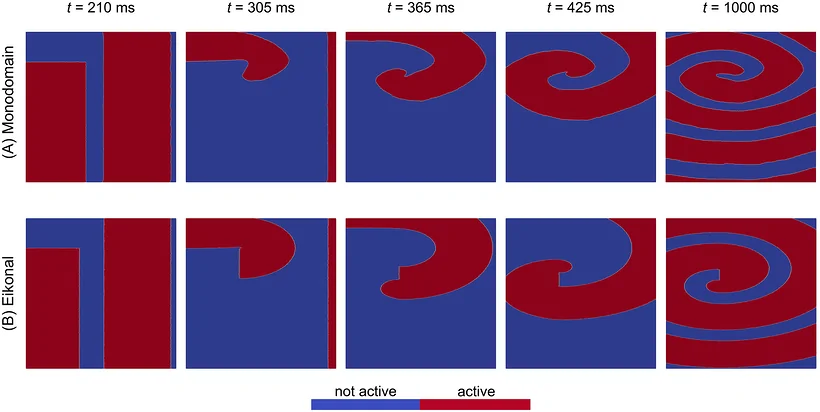
Spiral‐wave simulation *A*, monodomain and *B*, eikonal simulations of a spiral wave.

#### Atrial flutter

We now consider the case of the atrial surface illustrated in Fig. 8.

**Figure 8:**
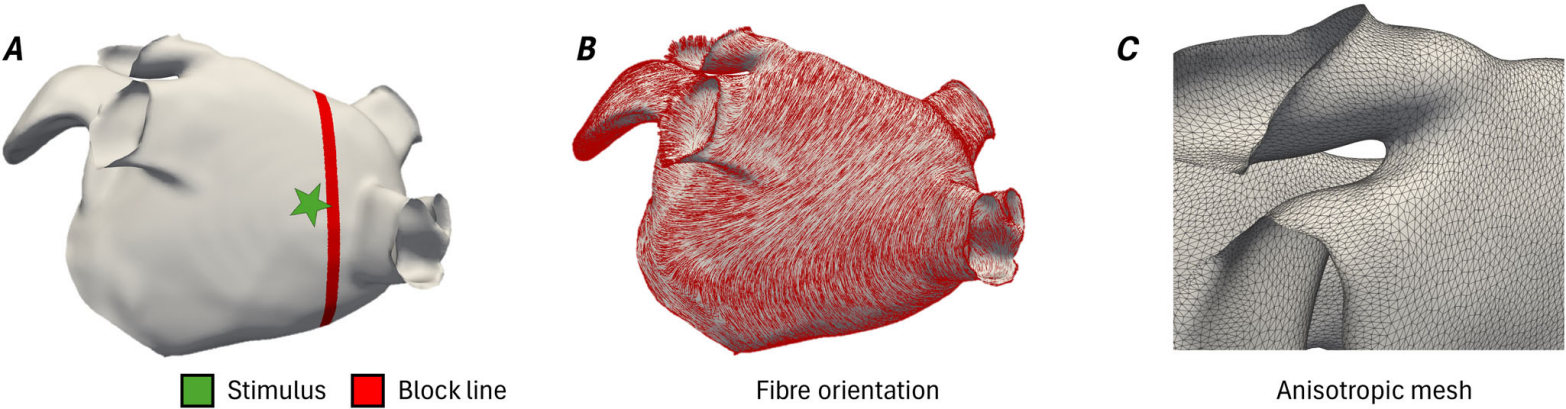
Left atrium geometry for the atrial flutter case *A*, the re‐entry is induced by a temporary block line in the proximity of the stimulus point. *B*, the anisotropic conduction is dictated by the fibre orientation. *C*, the adapted mesh follows the fibre orientation.

We compute the anisotropy ratio $CV_{t}/CV_{l}$ for the surface with a monodomain simulation as described in the Ventricular tachycardia section and motivated by the observations in Appendix, Anisotropy ratios. The longitudinal CV is 68cm/s, whereas the transversal CV is 27cm/s. Therefore, the anisotropy ratio is $CV_{t}/C\;V_{l}=0.4$. This result is in agreement with the formulas (Colli Franzone et al., 2014)

$$C\;V_{l}=\frac{\rho \sqrt{\sigma_{m}^{l}}}{C_{m}\sqrt{\beta }}\;\;\mathrm{and}\;C\;V_{t}=\frac{\rho \sqrt{\sigma_{m}^{t}}}{C_{m}\sqrt{\beta }}\;,$$

where ρ∈R is a parameter that depends on the ionic model. Indeed, $CV_{t}/C\;V_{l}=\sqrt{\sigma_{m}^{t}/\sigma_{m}^{l}}=0.4$. Again, the isotropic mesh for the monodomain simulation is not suitable for the eikonal simulation, as the percentage of triangles not satisfying the acuteness condition is 75.4%. Instead, the anisotropic mesh generated with the Mmg software has 8.5% of triangles that do not satisfy the acuteness condition and is more suitable. The number of nodes is approx. 47,500.

AFlut is initiated by a single stimulation with the block line approach (Gharaviri et al., 2020). The block line and the stimulation site are shown in Fig. 8. The stimulus is applied at time t=0ms. The block line is initially non‐conductive and becomes conductive at time t=125ms. The monodomain and the eikonal simulations are shown in Fig. 9.

**Figure 9:**
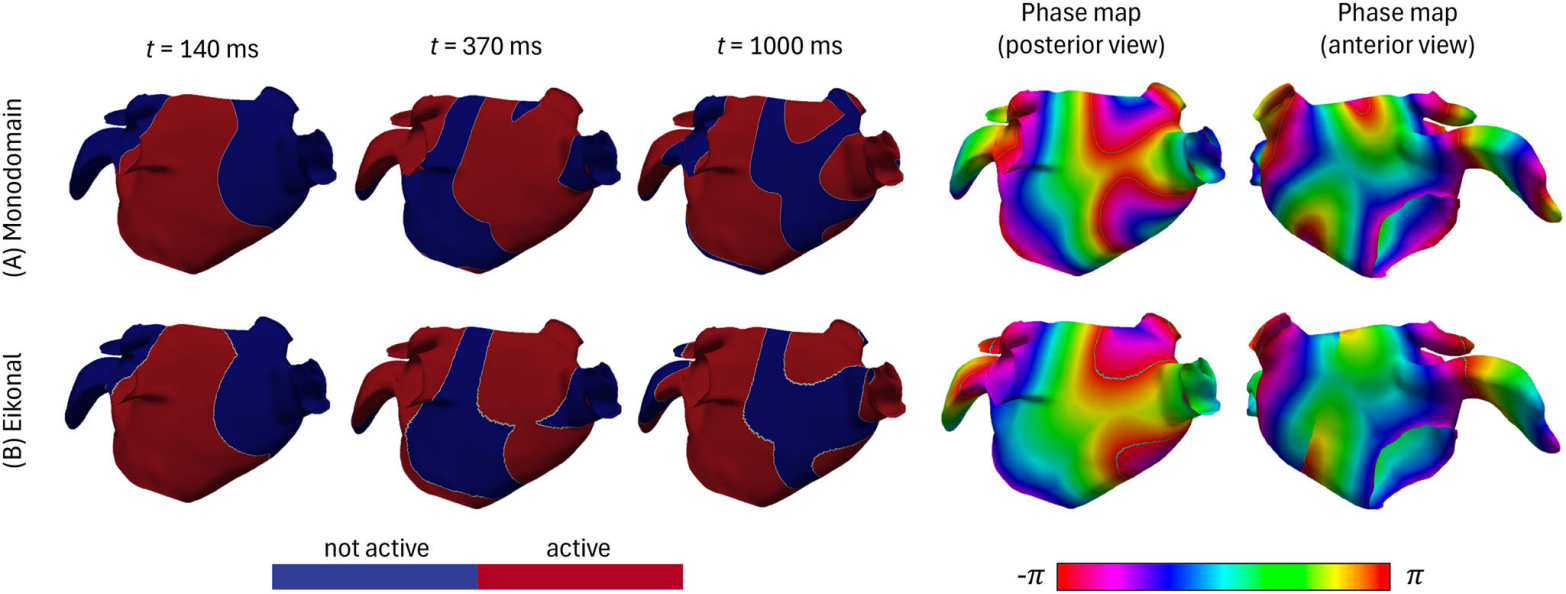
Re‐entry simulation on a patient‐specific left atrium and corresponding phase map for the monodomain model (*A*) and eikonal model (*B*) We note that the front is accurately captured by the eikonal model, except for a dispersion error and curvature effects. The phase maps for the dominant frequency show good agreement.

The action potential initially propagates upwards and towards the left. Time t=140ms is shortly after the block line became conductive and the action potential propagates also towards the right in the anterior wall. Around time t=230ms there is the emergence of two spiral waves with tips located in the posterior wall, one closer to the roof and the other on the opposite side, near the mitral ring. This re‐entrant dynamics repeats, as illustrated by the snapshots at times t=370ms and t=1000ms, and self‐sustains until the end of the simulation at time t=5000ms. Again, because the eikonal model does not capture the effect of the wavefront curvature, there are differences between the simulated re‐entries. The tip of the re‐entry is fixed in the eikonal simulation, whereas it moves in the monodomain simulation. More precisely, in the eikonal model there is no tip of the spiral, but rather a ‘singular’ segment in the phase map (rightmost panels of Fig. 9). As a consequence, the monodomain rotation is faster than the eikonal rotation. However, the eikonal propagation is faster than the monodomain propagation, since the action potential propagates as a convex front. The dominant frequency is similar in the two models, respectively 8.0 Hz in the monodomain and 9.0 Hz in the eikonal model. Similarly, the phase maps corresponding to the dominant frequency show good agreement both in posterior and anterior view.

## Discussion

In this work, we have presented a fast eikonal model with re‐excitability for simulating cardiac arrhythmias. The model is based on the FMM and incorporates the anisotropy through mesh adaptation. We validated its performance against the monodomain model in several numerical experiments, including two cases of clear clinical relevance. The resulting model is not intended to replace detailed biophysical simulations, but to provide a controlled way to separate the substrate for wave propagation from the restitution properties that determine when tissue can be re‐excited. In the examples considered here, re‐entry is sustained only when the anatomical pathway, conduction anisotropy and local recovery time are mutually compatible. This provides a mechanistic interpretation of the simulations: changes in conduction pathway or tissue recovery can be varied independently to assess whether they promote unidirectional block, re‐excitation and maintenance of macro‐re‐entry.

The eikonal model reproduced the main features of re‐entries rotating around scars, and was also able to reproduce focal re‐entries with meandering spiral core, showing good agreement with monodomain simulations. The differences observed between the two approaches are mainly due to the effect of the wavefront curvature (Fast & Kléber, 1997; Roney et al., 2026) on CV, which is not captured by the standard eikonal model.

The computational cost of the monodomain model depends on the size of the discretized linear system and the complexity of the ionic model. Instead, the computational cost of the eikonal model scales with the number of node updates. Therefore, the speedup varies depending on the numerical experiment. In our numerical experiments, our implementation of the eikonal model is between 7 and 10 times faster than the implementation of the monodomain model. Since the FMM has no numerical stability issues (Colli Franzone et al., 2014), it can use coarser meshes without introducing numerical instabilities, potentially leading to even larger gains in speed. Furthermore, our monodomain implementation was highly optimized and executed ionic models on a GPU, suggesting that the performance advantage of the eikonal approach is robust.

Our Python implementation of the FMM with re‐excitability is a prototype and not optimized for performance. For benchmarking, we compared it against other eikonal implementations. For the same setup, the C++‐based scikit‐fmm implementation required only 4ms, outperforming our unoptimized version (83.86s) and achieving better‐than‐real‐time performance. Note that the adaptations to the FMM required to include the re‐excitability do not change the computational complexity, which remains O(NlogN) (Kimmel & Sethian, 1998; Sethian & Vladimirsky, 2000). We therefore anticipate that an optimized C++ implementation would potentially achieve real‐time performance.

There is a compelling need for computational models based on imaging data that meet practical requirements when employed to non‐invasively study cardiac arrhythmias and plan their treatment. These requirements include a computational cost compatible with clinical time constraints and the ability to operate without off‐site high‐performance computing resources (Campos et al., 2022). The eikonal model with re‐excitability presented in this work meets these requirements, as it can operate on desktop computational resources and has the potential for near real‐time computation. In particular, the setup for ventricular or atrial tachycardia considered here could be used to quickly scan for re‐entry circuits (Bhagirath, Campos, et al., 2024) or to evaluate potential ablation strategies (Prakosa et al., 2018). By using the lead‐field approach (Grandits et al., 2025), our approach could be seamlessly integrated with surface electrocardiogram (ECG) simulation and Purkinje network integration (Álvarez‐Barrientos et al., 2025), paving the way towards non‐invasive assessment of ventricular and atrial tachycardia.

In fact, *in silico* ablation planning often needs the evaluation of the arrhythmia inducibility after pacing from several locations (Campos et al., 2022; Gander et al., 2022). Therefore, in such cases, it is worth investing the modest preprocessing effort required by the eikonal model, namely anatomical meshing, anisotropic adaptation and restitution‐curve computation, to exploit its near‐real‐time potential instead of repeatedly solving the monodomain model.

This distinction is particularly relevant in the context of CDTs. Purely data‐driven emulators and neural‐network surrogates can provide very fast inference, but their applicability is determined by the training set and by the variables chosen for parametrization, which may include patient anatomy, scar and fibrosis distribution, conduction properties, pacing protocol and selected outputs (Salvador et al., 2024; Yin et al., 2024). In contrast, the present model does not learn a patient‐specific input–output map from high‐fidelity simulations. It preserves an explicit physiological decomposition: geometry, anisotropy and non‐conductive regions define the propagation substrate, whereas restitution curves define local recovery and re‐excitability. The latter can be obtained from inexpensive 1‐D simulations or experimental restitution data and can be modified independently of the anatomical model. This structure makes the model transparent and modular: specific changes in tissue recovery or conduction can be tested for their effect on unidirectional block, re‐excitation and maintenance of macro‐re‐entry, while retaining the potential for near‐real‐time performance and preserving qualitative agreement with monodomain simulations in the clinically relevant macro‐re‐entry cases considered here.

In order to be applied to patient‐specific cases, the eikonal model needs a relatively inexpensive preprocessing phase. Similarly to the monodomain model, an anatomical twinning from imaging data is required to create the computational mesh and map tissue properties such as anisotropic conduction, scars and border zones. The mesh adaptation step is no more expensive than standard meshing; in our experiments, adaptation of the atrial mesh in Fig. 8 took 24 s on a laptop. Furthermore, the mesh adaptation depends only on the anisotropic ratio, and not the specific CV values. Additionally, the model requires computation of the restitution curves, which is negligible compared to the cost of performing several 3‐D monodomain simulations. The S1–S2 protocol is computationally effective because the state of the tissue immediately before the S2 stimulus can be stored and reloaded for different CLs.

In this work, we focused on 2‐D, triangulated surfaces. The FMM and the modifications to include the re‐excitability can straightforwardly be applied to 3‐D domains discretized by tetrahedra. The mesh adaptation to handle the anisotropy can be performed with the Mmg software (Dobrzynski & Frey, 2008) also on tetrahedral elements. However, since tetrahedra have more angles than triangles, in order to obtain a mesh with a high percentage of elements that satisfy the acuteness condition, one would need to increase the spatial resolution, thus increasing the computational cost. Importantly, the present surface formulation does not resolve transmural fibre rotation in full‐thickness myocardium. General 3‐D anisotropic mesh‐generation strategies have been developed and tested in a variety of metric fields (Rouxel‐Labbé, 2016), and therefore a full‐thickness extension is not precluded in principle. In a ventricular model, however, anisotropic remeshing would have to accommodate a fibre field that varies through the wall while preserving the metric‐acuteness constraints required by the FMM. This may require substantially finer tetrahedral meshes and could reduce the practical speed‐up. Extension to full‐thickness anatomies with transmural fibre rotation therefore remains an important limitation of the present work and a target for future developments.

A limitation of this work is the omission of fibrosis. Conduction discontinuities due to fibrotic tissue introduce delays in the activation times, which are exaggerated in the discretized monodomain model but not fully captured by the eikonal approximation (Gander et al., 2023). This limitation could be mitigated by employing the eikonal‐diffusion model (Colli Franzone et al., 2014), which reproduces conduction delays and curvature effects (Keener, 1991; Colli Franzone et al., 2014; Roney et al., 2026). Wavefront curvature influences both CV and action potential duration, although these effects become significant only at high curvature. The applicability of the eikonal‐diffusion and the eikonal‐curvature models to simulations of re‐entries needs to be studied in the future, in terms of both algorithmic inclusion of the re‐excitability and computational cost.

Taken together, these results demonstrate that the proposed eikonal model provides a physiologically grounded, computationally efficient framework for simulating cardiac arrhythmias. Its potential for near‐real‐time performance makes it a promising candidate for future applications in patient‐specific CDTs and non‐invasive therapy planning.

## Additional information

## Competing interests

None.

## Author contributions

L.G.: Conceptualization, methodology, software, validation, formal analysis, investigation, data curation, writing – original draft, writing – review and editing, visualization. F.S.C.: Methodology, resources, writing – original draft, writing – review and editing, funding acquisition. R.K.: Conceptualization, resources, writing – original draft, writing – review and editing, project administration, funding acquisition. S.P.: conceptualization, methodology, resources, writing – original draft, writing – review and editing, project administration, funding acquisition.

## Funding

This project has received funding from the European Research Council (ERC) under the European Union's Horizon Europe research and innovation programme (Grant agreement No. 101230597, awarded to SP). SP and RK acknowledge the support of the SNSF “CardioTwin” project (no. 214817). SP also acknowledges the PRIN‐PNRR project no. P2022N5ZNP, the INdAM‐GNCS, the MICROCARD‐2 project (project ID 101172576) and the CSCS‐Swiss National Supercomputing Centre project no. lp157. FSC acknowledges the support of the project ANID ‐ ERAPERMED‐13 and ANID ‐ Millennium Science Initiative Program ‐ ICN2021_004.

## Generative AI statement

ChatGPT 5.5 Instant (accessed May 2026) was used solely for proofreading and language editing of the final submitted R2 version of the manuscript. All manuscript content was written, reviewed and verified by the authors, who take full responsibility for the final text.

## Supporting information


Peer Review History

## Figures and Tables

**Figure 10:**
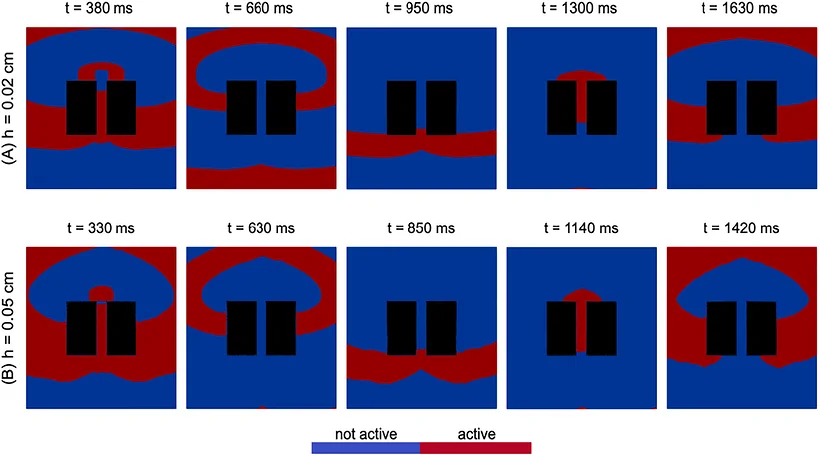
Effect of spatial resolution Monodomain simulations of a re‐entry in the presence of scars with space resolution *A*, h=0.02cm and *B*, h=0.05cm.

**Figure 11:**
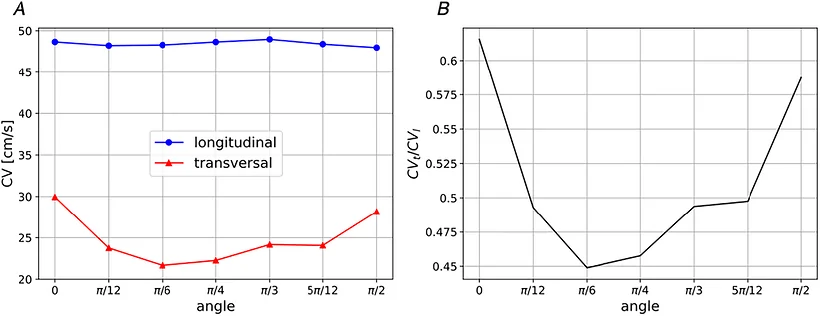
Longitudinal and transversal CVs (*A*) and anisotropy ratio (*B*) *versus* angle of the fibre orientation with respect to the horizontal axis The average edge length in this experiment is 0.05 cm.

## Data Availability

The full source code, input data and results is available in a public repository at the following address: https://github.com/HiddenHeartLab/reentry‐eikonal.

## Discretization issues

In the Re‐entries in the presence of scars and Spiral waves sections, the monodomain CVs are affected by the spatial discretization. In this section we analyse the influence of the spatial discretization on the monodomain simulations and on the anisotropy ratios.

### Monodomain simulations

The space resolution h=0.05cm used in the sections re‐entries in the presence of scars and spiral waves affects the monodomain simulations. As a comparison, we perform the monodomain simulation of the 2‐D case in the Re‐entries in the presence of scars section with the finer resolution h=0.02cm. The comparison is shown in Fig. 10. Since we use finite elements, the finer spatial discretization has the effect of reducing the CV (Pezzuto et al., 2016). Therefore, in Fig. 10, the two simulations are illustrated by snapshots taken at different times.

In the simulation with the finer resolution, the ellipsoidal shape of the depolarization and repolarization fronts is more pronounced than in the simulation with the coarser resolution. In fact, when the mesh is too coarse, the front shape is affected by the mesh topology. Contrary to the monodomain model, the eikonal model captures the ellipsoidal shape of the fronts also at resolution h=0.05cm (see Fig. 4). Note that the same observations also hold for the 2‐D spiral‐wave simulations shown in Fig. 7.

### Anisotropy ratios

The spatial discretization affects the monodomain CVs and, as a consequence, the anisotropy ratios. To illustrate this, we consider the isotropic triangulated mesh used in the 2‐D re‐entry and spiral‐wave cases, and fibre orientations with variable angles with respect to the horizontal axis. For all the fibre directions, we simulate a monodomain propagation with the Mitchell–Schaeffer ionic model for the healthy ventricular tissue and we compute the longitudinal and transversal CVs and the corresponding anisotropy ratios. The results are shown in Fig. 11 for seven angles varying between 0 and π/2.

The longitudinal CV is approximately constant, whereas the transversal CV, and consequently the anisotropy ratio, is larger for horizontal and vertical fibres than for diagonal fibres. Since the coarser spatial discretization has the effect of increasing the CV (Pezzuto et al., 2016), these results suggest that the triangles of the mesh are mostly oriented along the horizontal and vertical axes, thus implying a coarser resolution along these directions. On a mesh discretizing a surface, it is unlikely that the fibre direction matches the orientation of the triangles. Therefore, it is appropriate to set diagonal fibres when approximating the CVs on a mesh discretizing a square. This motivates our choice for the computation of the anisotropy ratios in the ventricular tachycardia and atrial flutter cases. Finally, it is interesting to note that the error is not symmetric with respect to π/4. In general, since the solution is mesh‐dependent at a coarse resolution, as in this case, if the mesh is not π/4‐symmetric (which it is not, being a triangular mesh), the solution will not be symmetric either.
