# Supplementary material for: An eikonal model with re‐excitability for fast simulations in cardiac electrophysiology
Source: J Physiol. 2026 Sep 1;604(19):8264–83. doi: 10.1113/JP287281 (PMC13628559; doi:10.1113/JP287281)
Supplement: Supplementary file 1 [file TJP-604-8264-s001.pdf]

The Journal of Physiology

An eikonal model with re-excitability for fast simulations in cardiac electrophysiology

Lia Gander, Rolf Krause, Francisco Sahli Costabal, and Simone Pezzuto
DOI: 10.1113/JP287281

Corresponding author(s): Simone Pezzuto (simone.pezzuto@unitn.it)

The referees have opted to remain anonymous.

Review Timeline:

Submission Date:	29-Oct-2024
Editorial Decision:	02-Jan-2025
Revision Received:	31-Oct-2025
Editorial Decision:	25-Nov-2025
Revision Received:	02-Jun-2026
Accepted:	10-Jul-2026

Senior Editor: Natalia Trayanova

Reviewing Editor: Christian Vergara

Transaction Report:

(Note: With the exception of the correction of typographical or spelling errors that could be a source of ambiguity, letters and reports are not edited. Depending on transfer agreements, referee reports obtained elsewhere may or may not be included in this compilation. Referee reports are anonymous unless the Referee chooses to sign their reports.)

2 October 2025

Dear Dr Pezzuto

We are starting to draw together all the articles for our 'Computational modelling of the cardiovascular system' special issue in the Journal of Physiology.

We must now issue a firm deadline of 31 October for submission of your revised article below, if you wish to be included in the special issue.

If you submit after this date, we can still consider your revised article, but we will no longer be able to guarantee its inclusion in the special issue (it would need to be a standalone item, in a regular issue).

We very much hope you can submit in time!

Best wishes

Diana
JP

5 June 2025

Just a gentle reminder that we are looking forward to receiving your revised version soon.
Best wishes
Diana
JP

Dear Dr Pezzuto,

Re: JP-RP-2024-287281 "An eikonal model with re-excitability for fast simulations in cardiac electrophysiology" by Lia Gander, Rolf Krause, Francisco Sahli Costabal, and Simone Pezzuto

Thank you for submitting your manuscript to The Journal of Physiology. It has been assessed by a Reviewing Editor and by 2 expert referees and we are pleased to tell you that it is potentially acceptable for publication following satisfactory major revision.

Please advise your co-authors of this decision as soon as possible.

The referee reports are copied at the end of this email.

Please address all the points raised and incorporate all requested revisions or explain in your Response to Referees why a change has not been made. We hope you will find the comments helpful and that you will be able to return your revised manuscript within 2 months. If you require longer than this, please contact journal staff: jp@physoc.org. Please note that this letter does not constitute a guarantee for acceptance of your revised manuscript.

Your revised manuscript should be submitted online using the link in your Author Tasks: https://jp.msubmit.net/cgi-bin/main.plex

If you do not wish to submit a revised version of your manuscript, you must inform our journal staff (jp@physoc.org) or reply to this email to request withdrawal. Please note that a manuscript must be formally withdrawn from the peer review process at one journal before it may be submitted to another journal.

TRANSPARENT PEER REVIEW POLICY: To improve the transparency of its peer review process, The Journal of Physiology publishes online as supporting information the peer review history of all articles accepted for publication. Readers will have access to decision letters, including Editors' comments and referee reports, for each version of the manuscript, as well as any author responses to peer review comments. Referees can decide whether or not they wish to be named on the peer review history document.

ABSTRACT FIGURES: Authors are expected to use The Journal's premium BioRender account to create/redraw their Abstract Figures. Information on how to access this account is here:
https://physoc.onlinelibrary.wiley.com/journal/14697793/biorender-access.

This will enable Authors to create and download high-resolution figures. If authors have used the free BioRender service, they can use the instructions provided in the link above to download a high-resolution version suitable for publication.

The link provided should only be used for the purposes of this submission. Authors will be charged for figures created on this account if they are not related to this manuscript submission.

LANGUAGE EDITING AND SUPPORT FOR PUBLICATION: If you would like help with English language editing, or other article preparation support, Wiley Editing Services offers expert help, including English Language Editing, as well as translation, manuscript formatting, and figure formatting at www.wileyauthors.com/eeo/preparation. You can also find resources for Preparing Your Article for general guidance about writing and preparing your manuscript at www.wileyauthors.com/eeo/prepresources.

RIGOUR AND REPRODUCIBILITY: We recommend authors consult The Journal's Rigour and Reproducibility requirements to ensure that their manuscript is suitable for publication: https://physoc.onlinelibrary.wiley.com/pb-assets/hub-assets/physoc/documents/TJP-Rigour-and-Reproducibility-Requirements.pdf

REVISION CHECKLIST:

Check that your Methods section conforms to journal policy: https://jp.msubmit.net/cgi-bin/main.plex

Check that data presented conforms to the statistics policy: https://jp.msubmit.net/cgi-bin/main.plex

Upload a full Response to Referees file. To create your 'Response to Referees': copy all the reports, including any comments from the Senior and Reviewing Editors, into a Microsoft Word, or similar, file and respond to each point, using font or background colour to distinguish comments and responses and upload as the required file type.

Please upload two versions of your manuscript text: one with all relevant changes highlighted and one clean version with no changes tracked. The manuscript file should include all tables and figure legends, but each figure/graph should be uploaded as separate, high-resolution files.

You may also upload:

- 'Potential Cover Art' for consideration as the issue's cover image
- Appropriate Supporting Information (Video, audio or data set: see https://jp.msubmit.net/cgi-bin/main.plex

We look forward to receiving your revised submission.

If you have any queries, please reply to this email and we will be pleased to advise.

Yours sincerely,

Natalia Trayanova
Senior Editor
The Journal of Physiology

REQUIRED ITEMS

- Include a Key Points list in the article itself, before the Abstract.

- Author photo and profile. First or joint first authors are asked to provide a short biography (no more than 100 words for one author or 150 words in total for joint first authors) and a portrait photograph. These should be uploaded and clearly labelled together in a Word document with the revised version of the manuscript. See Information for Authors for further details.

- The reference list must be in alphabetical order, rather than numbered, to comply with our Journal format.

- Your manuscript must include a complete Additional Information

section, including competing interests; funding; author contributions and acknowledgements.

- Please upload separate high-quality figure files via the submission form.

- Please ensure that the Article File you upload is a Word file.

- A Data Availability Statement is required for all papers reporting original data. This must be in the Additional Information section of the manuscript itself. It must have the paragraph heading 'Data Availability Statement'. All data supporting the results in the paper must be either: in the paper itself; uploaded as Supporting Information for Online Publication; or archived in an appropriate public repository. The statement needs to describe the availability or the absence of shared data. Authors must include in their statement: a link to the repository they have used, or a statement that it is available as Supporting Information; reference the data in the appropriate sections(s) of their manuscript; and cite the data they have shared in the References section. Whenever possible, the scripts and other artefacts used to generate the analyses presented in the paper should also be publicly archived. If sharing data compromises ethical standards or legal requirements then authors are not expected to share it, but must note this in their statement. For more information, see our Statistics Policy.

- Please include an Abstract Figure file, as well as the Figure Legend text within the main article file. The Abstract Figure is a piece of artwork designed to give readers an immediate understanding of the research and should summarise the main conclusions. If possible, the image should be easily 'readable' from left to right or top to bottom. It should show the physiological relevance of the manuscript so readers can assess the importance and content of its findings. Abstract Figures should not merely recapitulate other figures in the manuscript. Please try to keep the diagram as simple as possible and without superfluous information that may distract from the main conclusion(s). Abstract Figures must be provided by authors no later than the revised manuscript stage and should be uploaded as a separate file during online submission labelled as File Type 'Abstract Figure'. Please also ensure that you include the figure legend in the main article file. All Abstract Figures should be created using BioRender. Authors should use The Journal's premium BioRender account to export high-resolution images. Details on how to use and access the premium account are included as part of this email.

EDITOR COMMENTS

Reviewing Editor:

The Referees ask you to fix several critical points. Please carefully answer to their remarks by preparing a detailed letter indicating where changes occur and by highlighting in color in the text such changes.

I have also a couple of personal comments, whose answers should be included in the letter:

Editor comments:

1) Did the authors used for their simulation in realistic geometries new patients or take them from the litearture or previous works. In the latter case, please provide suitable references. In the former case, please specify if:

- An informed consent has been obtained, preferably in writing

- Studies are conformed to the standards set by the Declaration of Helsinki

- The procedures have been approved by the local ethics committee and the appropriate reference number has been stated

2) Referee 2 is not sure that the paper is suitable for the journal: "the manuscript appears to be more appropriate for a numerical methods journal rather than the Journal of Physiology." The authors should convince the suitability of the paper

for this journal. Please add sentences and discussions that highlight that the methods and the results are relevant non only for a journal of numerical methods.

Please also see 'Required Items' above.

Senior Editor:

This paper does not have a major physiology component to be appropriate for this journal. In addition, the comments and criticisms are significant. While a major revision is suggested at this round, unless the paper includes significant physiological insight and addresses all the reviewers comments on the next round, the paper will not be considered further.

REFEREE COMMENTS

Referee #1:

See attached file.

Referee #2:

Please see the attached file.

END OF COMMENTS

This paper describes a method to quickly compute propagation in cardiac tissue. It uses the Eikonal method which has been used previously but adds reexcitability based on diastolic interval. The technique is of interest to those wishing to reduce the time to compute propagation simulations, such as for clinical applications. The paper is straightforward and the results are reasonable and what one would expect. The paper is logically organized.

The Eikonal equations given do not provide the boundary condition, which is ignored by the FMM.

The condition of acute triangles seems unique to the authors' formulation.
Does remeshing need only to be done once, regardless of the excitation pattern?

It would have greatly strengthened the paper to have a 3D implementation since it seems relatively simple to extend. How would performance metrics change in 3D?

No reentry simulations will ever agree since the system is chaotic and the smallest differences grow exponentially over time. However, the simulations should have the same statistical properties. So, when comparing functional reentries, the authors shoud allow the simulations to progress long enough to compare period, rotor center, spiral tip meander, etc.

The authors compute APD restitution curves in 0D. They may not necessaily match those in 3D. Have the authors tested this?

For the Appendix II, what mesh resolution is used? Also, should it not be symmetric about pi/4?

Disclaimer: This is a confidential document.

The manuscript describes an algorithm based on the eikonal model that accounts for tissue re-excitability and is suitable for simulating re-entries in cardiac electrophysiology. In this regard, the reviewer appreciates the limited physiological content in the manuscript. However, the manuscript appears to be more appropriate for a numerical methods journal rather than the *Journal of Physiology*. Aside from this consideration, there are several major comments that the authors should address before the manuscript can be considered.

Major Points:

1. The results are compared only qualitatively, even though specific quantification is possible. In fact, the authors acknowledge this in the abstract. For instance, they could report the difference in dominant frequency between the results obtained with the monodomain model and those obtained with the proposed algorithm. When modeling arrhythmias, it is important to reproduce key features in the simulation, such as the patterns and frequency of re-entry. Moreover, a discretization of 0.05 cm for the monodomain model with the Courtemanche model may not be very accurate. In atrial models using this ionic model, a discretization of 0.025 cm is typically used.
2. One major limitation identified by this reviewer is that the model does not account for electrotonic coupling, which modulates the action potential duration of neighboring cells with different electrophysiological properties, particularly at the boundaries of regions. This phenomenon is significant in highly heterogeneous tissues, such as the atrium. Additionally, APD restitution curves were computed using a 0D cell model. However, the same 1D simulations used to compute the restitution of conduction velocity could also be used to compute APD restitution. It is well established that APD restitution computed in fibers differs from that computed in isolated cells due to the presence of electrotonic coupling. Since this is primarily a methodological paper, the authors should perform simulations using APD restitution properties obtained from fibers and compare these results with their current findings.
3. Regarding the restitution protocol, the authors employ a somewhat unconventional S1-S2 protocol compared to those typically used in clinical or in-vitro settings. The classical S1-S2 protocol involves delivering 10 stimuli at a basic cycle length (BCL), followed by one stimulus at a given coupling interval (CL), repeating the process at progressively shorter coupling intervals until conduction block occurs. Why did the authors choose this alternative protocol? Furthermore, the nature of the method suggests that a dynamic restitution curve would be more appropriate than the S1-S2 protocol. Dynamic restitution provides insights into alternans, which commonly occur at lower CL values—a highly pro-arrhythmic condition.
4. Conduction velocity (CV) restitution curves were computed for a given mesh size and tissue conductivity. However, for a specific tissue conductivity, the numerical CV restitution curve is highly sensitive to mesh size. I believe the authors computed the CV restitution curve using a very fine mesh (0.05 mm, which is quite fine) to ensure it approximates the true conduction velocity restitution for the given tissue conductivity. Please make this explicit in the text.
5. All the examples presented only show cases of single-rotor re-entries. However, clinically relevant problems, such as atrial fibrillation, are not demonstrated using the present methodology. The authors must demonstrate the capability of the methodology to reproduce multi-front re-entries to illustrate its true potential for clinical applications.

6. Another major concern is the handling of tissue anisotropy. To address anisotropy, the algorithm must work with anisotropic meshes. The examples shown in the manuscript are all 2D, i.e., rectangular patches and shell models of the ventricle and atria, with simplified and unrealistic fiber orientations (e.g., circumferential for the ventricle and projections of the (1,0,0) unit vector onto the atrial surface). In reality, fiber orientation varies by position—transmurally in the ventricle and regionally in the heart. Generating anisotropic meshes in such cases is challenging, particularly for three-dimensional problems, as well as in the atria, where fiber orientation changes from region to region. This poses a significant limitation to the method.

Minor Points:

1. In the abstract, the authors state: "... the monodomain model is computationally costly and has limited applicability in practice." While it is true that the monodomain model is a simplification of the bidomain model, it remains widely used by the modeling community to describe cardiac electrophysiology, as it accommodates detailed electrophysiological models of cardiac cells. Considering that the Eikonal model only provides activation times and does not incorporate detailed cellular electrophysiology (or includes information limited to restitution behavior), it is even more limited than the monodomain model. Please reword this statement or consider removing it.
2. In the third line of the abstract: "Thus, it desirable ..." should be corrected to "Thus, it is desirable ...".
3. In Algorithm 1, the terms $N(X_0)$ and $HL(.)$ are not explained. I believe *HL* stands for the Hopf-Lax formula. Please clarify this in the manuscript.
4. In Algorithm 2, the terms $a(.)$ and $c(.)$ are not defined. The nomenclature must be clearly described in the text for better understanding.
5. For completeness, it would be helpful to include a figure—at least in the appendix—showing the actual fiber direction and the mesh used in the computations.

An eikonal model with re-excitability for fast simulations in cardiac electrophysiology

Lia Gander, Rolf Krause, Francisco Sahli Costabal, Simone Pezzuto

Reviewing Editor

The Referees ask you to fix several critical points. Please carefully answer to their remarks by preparing a detailed letter indicating where changes occur and by highlighting in color in the text such changes.

I have also a couple of personal comments, whose answers should be included in the letter:

1. Did the authors used for their simulation in realistic geometries new patients or take them from the litearture or previous works. In the latter case, please provide suitable references. In the former case, please specify if:
 - An informed consent has been obtained, preferably in writing
 - Studies are conformed to the standards set by the Declaration of Helsinki
 - The procedures have been approved by the local ethics committee and the appropriate reference number has been stated

Thanks for noticing, we added the following section on *Clinical data* to the manuscript.

> *We considered two patient-specific models: a case of a left ventricle with a scar in the septal region, and a bi-atrial model. Both are based on clinical data previously described.*
>
> *The ventricular model is one case of a cohort of patients who underwent cardiac resynchronization therapy [1, 2]. The institutional review board approved the study protocol, and all patients gave written and oral informed consent for the investigation. The study was performed in compliance with the Declaration of Helsinki. The anatomical model (heart, torso, lungs, major blood masses) were reconstructed from cardiac magnetic resonance (CMR) scan with late gadolinium-enhancement (LGE). Surfaces of relevant anatomical structures were constructed from their contours using Blender (The Blender Foundation). The outline of the ventricles was detected from short-axis cine images. Scarred tissue and border zone was inferred from LGE-MRI sequence by manual contour and alignment. Ventricular fiber orientation was assigned using a rule-based approach [3]. The left-ventricular surface model has been based on the mid-myocardium of the full 3-D model. The valvular plane has been closed to facilitate the re-entry.*
>
> *The atrial model has also been previously described [4] and openly accessible on Zenodo [5]. Specifically, we selected case "P1", only the left atrium. We modified the original bilayer by extracting only the endocardium. We used the Mmg software [6] to refine the original mesh, obtaining a grid with average edge size of* $0.02\,\mathrm{cm}$ *and approx.* $213\,000$ *nodes. The fiber, sheet and tag fields have been interpolated from the original mesh onto the refined one with radial basis functions, as implemented in the software* `pyvista`.

2. Referee 2 is not sure that the paper is suitable for the journal: "the manuscript appears to be more appropriate for a numerical methods journal rather than the Journal of Physiology." The authors should convince the suitability of the paper for this journal. Please add sentences and discussions that highlight that the methods and the results are relevant non only for a journal of numerical methods.

Thanks for pointing this out. We significantly revised Abstract, Introduction and Discussion to better frame the importance of fast and clinically feasible computational models in the study of cardiac arrhythmias.

3. Please also see "Required Items" above.

Fixed.

Senior Editor - Comments

This paper does not have a major physiology component to be appropriate for this journal. In addition, the comments and criticisms are significant. While a major revision is suggested at this round, unless the paper includes significant physiological insight and addresses all the reviewers comments on the next round, the paper will not be considered further.

We thank the editor for the opportunity to revise our manuscript and strengthen its physiological focus. All reviewer comments have been thoroughly addressed, and the Abstract, Introduction, and Discussion have been substantially rewritten to emphasize the physiological relevance of our approach to cardiac arrhythmias and its integration within digital twin frameworks.

We are also grateful to the reviewers for their constructive feedback, which has helped us substantially improve the clarity and physiological framing of the manuscript.

Reviewer #1 - Comments

This paper describes a method to quickly compute propagation in cardiac tissue. It uses the Eikonal method which has been used previously but adds reexcitability based on diastolic interval.

The technique is of interest to those wishing to reduce the time to compute propagation simulations, such as for clinical applications. The paper is straightforward and the results are reasonable and what one would expect. The paper is logically organized.

Many thanks for the positive feedback.

1. The Eikonal equations given do not provide the boundary condition, which is ignored by the FMM.

We believe our formulation is correct. In Algorithm 2, we apply the stimulus exactly by setting a boundary condition for the eikonal equation. On the other hand, points that are refractory will not trigger a propagation (those labelled as *Accepted*).

2. The condition of acute triangles seems unique to the authors' formulation. Does remeshing need only to be done once, regardless of the excitation pattern?

Exactly, as mentioned in the Discussion, the remeshing only depends on the local anisotropic ratio, and not the conduction velocity, which instead can vary (as it does due to restitution properties.) It is also not particularly expensive: in a surface atrial model with 110k nodes, remeshing takes approximately 24 s.

3. It would have greatly strengthened the paper to have a 3D implementation since it seems relatively simple to extend. How would performance metrics change in 3D?

Thanks for pointing this out. We fully agree that the 3-D extension would be extremely interesting. The impact on the performance is due to the following aspects: 1) the local solver (the Hopf-Lax update) is more expensive, because we need to solve a higher-dimensional optimization problem; 2) each node has more neighbors than in 2-D, usually 4 times more; 3) a 3-D geometry has more nodes. Note however that this also applies in a similar manner to the monodomain equation, therefore we should still retain a significant speed-up.

4. No reentry simulations will ever agree since the system is chaotic and the smallest differences grow exponentially over time. However, the simulations should have the same statistical properties. So, when comparing functional reentries, the authors shoud allow the simulations to progress long enough to compare period, rotor center, spiral tip meander, etc.

We fully agree with the reviewer. Besides chaos, also in a stable reentry we would observe a divergence of the error because of slightly different numerical conduction velocity and restitution.

We performed a comparison in terms of phase map and dominant frequency analysis, to better quantify the error, in the most complex case of atrial simulation. We added the following:

> *The dominant frequency is similar in the two models, respectively* 8.0 Hz *in the monodomain and* 9.0 Hz *in the eikonal model. Similarly, the phase maps corresponding to the dominant frequency show good agreement both in the posterior and anterior view.*

5. The authors compute APD restitution curves in 0D. They may not necessaily match those in 3D. Have the authors tested this?

Thanks for noticing. We recomputed the restitution properties in a 1-D propagation, together with conduction velocity, which were already computed with the cable equation. We fully agree that a 1-D setup is more appropriate because in a single cell case the stimulation current may affect the APD. In our case, differences were minimal. Finally, compared to a 3D propagation, we are not considering here the effect of curvature, that we covered in another work [7].

6. For the Appendix II, what mesh resolution is used? Also, should it not be symmetric about $\pi/4$?

Thanks for raising this interesting point. We added to caption that the mesh size was 0.05 cm, therefore relatively coarse for a monodomain simulation. For the symmetry, we added the following sentence:

> *Finally, it is interesting to note that the error is not symmetric with respect to $\pi/4$. In general, since the solution is mesh-dependent at coarse resolution, such as this case, if the mesh is not $\pi/4$-symmetric (which is not, being a triangular mesh), the solution will not be symmetric either.*

Reviewer #2 - Comments

The manuscript describes an algorithm based on the eikonal model that accounts for tissue re-excitability and is suitable for simulating re-entries in cardiac electrophysiology. In this regard, the reviewer appreciates the limited physiological content in the manuscript. However, the manuscript appears to be more appropriate for a numerical methods journal rather than the Journal of Physiology. Aside from this consideration, there are several major comments that the authors should address before the manuscript can be considered.

We thank the reviewer for this valuable feedback and for highlighting the need to better emphasize the physiological relevance of our work. We have carefully revised the manuscript to address this concern.

In particular, we expanded the Abstract, Introduction and Discussion to clearly position our contribution within the study of cardiac arrhythmias, emphasizing its potential applications in patient-specific digital twin technologies and non-invasive therapy planning. The revised text now highlights how the proposed model captures key physiological phenomena, such as anisotropic conduction, re-entry formation, and tissue re-excitability, while enabling near real-time simulations relevant for clinical decision support. We believe these changes clarify the physiological motivation and significance of our approach, aligning the manuscript more closely with the scope of the Journal of Physiology.

Major Points:

1. The results are compared only qualitatively, even though specific quantification is possible. In fact, the authors acknowledge this in the abstract. For instance, they could report the difference in dominant frequency between the results obtained with the monodomain model and those obtained with the proposed algorithm. When modeling arrhythmias, it is important to reproduce key features in the simulation, such as the patterns and frequency of re-entry. Moreover, a discretization of 0.05 cm for the monodomain model with the Courtemanche model may not be very accurate. In atrial models using this ionic model, a discretization of 0.025 cm is typically used.

Thanks for your suggestions, we now added an more quantitative analysis for the atrial model. Specifically, we computed the dominant frequency and the corresponding phase map in monodomain and eikonal models. We added the following text:

> *The dominant frequency is similar in the two models, respectively 8.0 Hz in the monodomain and 9.0 Hz in the eikonal model. Similarly, the phase maps corresponding to the dominant frequency show good agreement both in the posterior and anterior view.*

We also extended the simulation up to 5 s, and refined the atrial geometry for the monodomain simulation to an average edge length of 0.02 cm. The Figures have been updated accordingly.

2. One major limitation identified by this reviewer is that the model does not account for electrotonic coupling, which modulates the action potential duration of neighboring cells with different electrophysiological properties, particularly at the boundaries of regions. This phenomenon is significant in highly heterogeneous tissues, such as the atrium. Additionally, APD restitution curves were computed using a 0D cell model. However, the same 1D simulations used to compute the restitution of conduction velocity could also be used to compute APD restitution. It is well established that APD restitution computed in fibers differs from that computed in isolated cells due to the presence of electrotonic coupling. Since this is primarily a methodological paper, the authors should perform simulations using APD restitution properties obtained from fibers and compare these results with their current findings.

Thanks for raising this important point. First of all, we recomputed the APD restitution properties with the cable equation, although differences were minor with respect to the 0-D case.

Regarding the electrotonic coupling, we fully agree with the analysis, and in fact this was already mentioned in the Discussion. In particular, we recently studied both the effect of curvature [7] and the effect of source-sink mismatch [8].

For the former, besides the well-known linear correction of the conduction velocity with respect to the anisotropic curvature, also the APD is modulated by the curvature. However, the modulation is appreciable only at high curvature.

Concerning the source-sink mismatch in the presence of heterogeneities in CV, we showed that the eikonal-diffusion model is very accurate in reproducing the phenomenon, and we derived a formula to determine the delay in activation depending on the jump in coefficients. (The eikonal-curvature model, on the other hand, cannot capture such an effect.) In our experience, however, the source-sink mismatch observed in monodomain simulations is greatly emphasized by a coarse numerical discretization [8, Fig. 4]. For instance, the delay introduced by a moderate jump in conductivity (say $\sigma_1 \approx 0.3\sigma_2$) across a discontinuity is of the order of 0.2 ms, but in a monodomain simulation with mesh resolution of 0.4 mm, still very common, can be much larger than 5 ms or even induce an artificial conduction block.

3. Regarding the restitution protocol, the authors employ a somewhat unconventional S1-S2 protocol compared to those typically used in clinical or in-vitro settings. The classical S1-S2 protocol involves delivering 10 stimuli at a basic cycle length (BCL), followed by one stimulus at a given coupling interval (CL), repeating the process at progressively shorter coupling intervals until conduction block occurs. Why did the authors choose this alternative protocol? Furthermore, the nature of the method suggests that a dynamic restitution curve would be more appropriate than the S1-S2 protocol. Dynamic restitution provides insights into alternans, which commonly occur at lower CL values—a highly pro-arrhythmic condition.

Thanks for raising this interesting point. We analyzed this aspect in depth, and we report below the results.

In simple 2-variables models such as Mitchell-Schaeffer, FitzHugh-Nagumo, Roger-McCulloch, and others, S1-S2 and dynamic pacing protocols should provide the same restitution curve. The proof is based on the same argument sketched in [9, Sec 3.1] using the fast-slow approximation.

In more physiological models, such as the Courtemanche model [10], the two approaches yield generally different restitution curves, as shown in Fig. 1. Specifically for the Courtemanche model, we observed a least 3 different time scales: the fast scale of the AP (order of 1 s), an intermediate scale of intracellular calcium dynamics (order of 1 min), and a slow scale for intracellular concentration of potassium and sodium (order of 1 h). Those scales can also be seen in the APD adaptation over long simulations. The S1-S2 restitution curve does not capture the intermediate and slow scales, thus it may sensibly differ from the curve obtained with a dynamic protocol. Interestingly, the slow scale affects quite significantly the APD in the Courtemanche model, which explains most of the differences between the curves. In fact, in comparing the two curves in other models (ten Tusscher 2004, for instance) we noticed a much less pronounced difference.

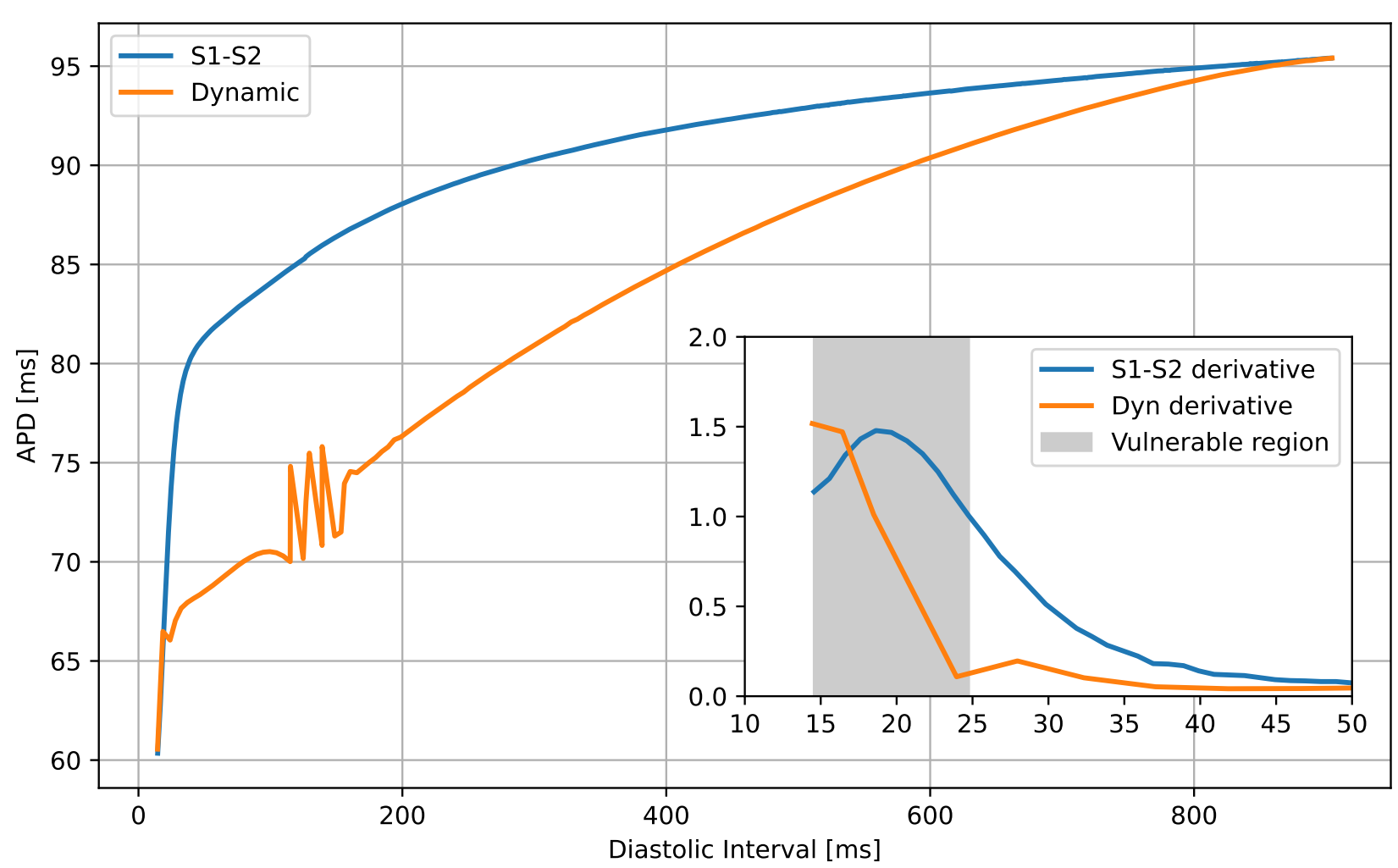

Figure 1: Comparison of the restitution curves obtained with S1-S2 protocol (blue) or with Dynamic protocol (orange), for the Courtemanche model. In the inset plot shows the derivative of the S1-S2 curve, highlighting its vulnerable region. The oscillatory region in the Dynamic curve is probably due to numerical instabilities in the Courtemanche model.

On the other hand, in a normal monodomain simulation is more likely to see the effect of the S1-S2 restitution, since we rarely simulate several minutes of arrhythmic events. Furthermore, the Courtemanche model (and several others) are probably not indicated for such long simulations.

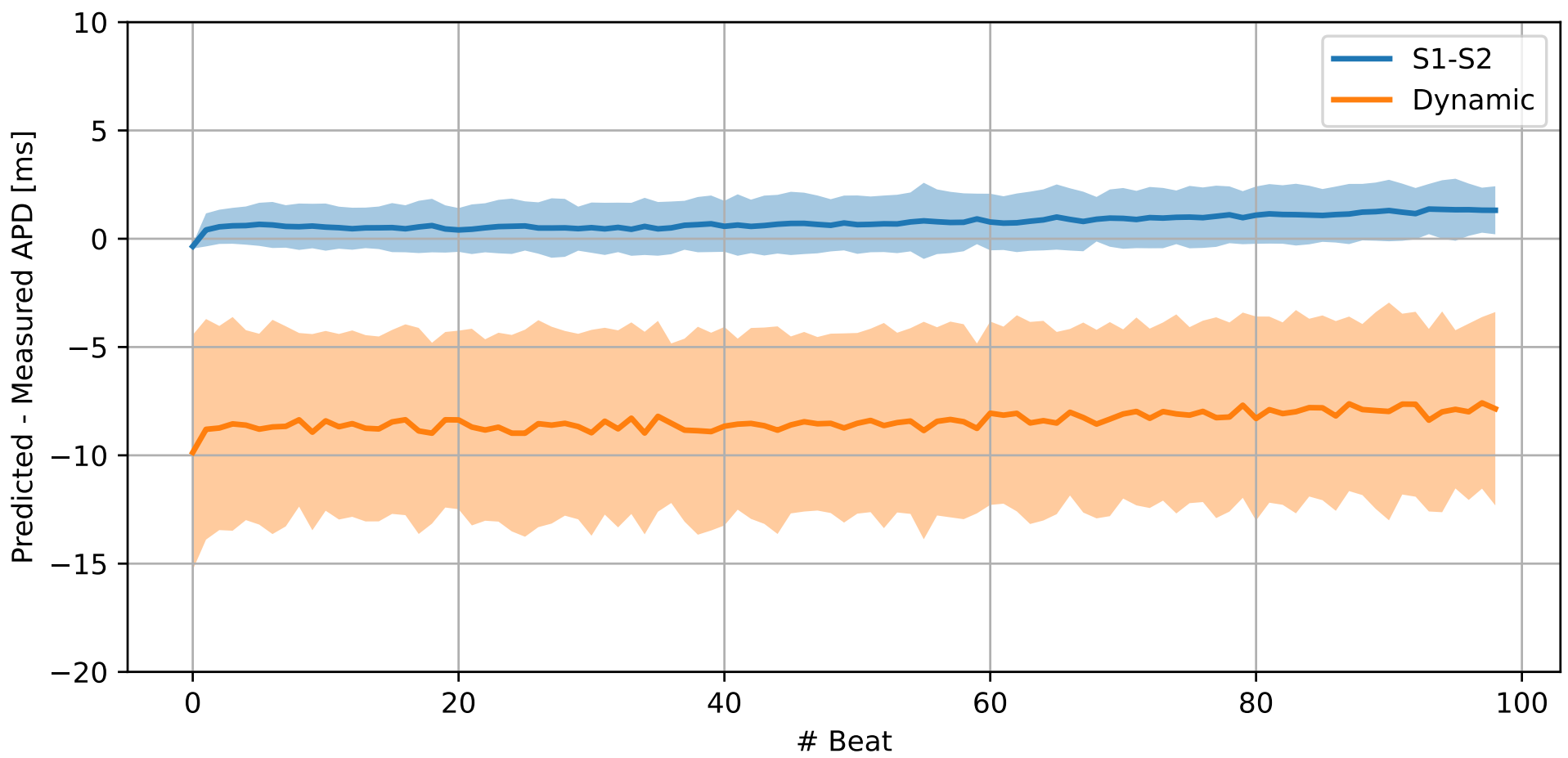

Figure 2: Comparison between S1-S2 restitution and dynamic restitution in predicting the APD in a random train of stimuli, with cycle length sampled uniformly between 150 ms and 500 ms. The dynamic restitution underestimates the APD.

In this respect, we prepared the following experiment. We computed the APD restitution curves using either a S1-S2 protocol or a dynamic protocol, using the Courtemanche model. Next, we simulated a pacing protocol with a train of $P = 100$ stimuli randomly selected from a uniform distribution between 150 ms and 500 ms. The experiment was executed with either the Courtemanche model or by using the restitution curves. The measured and predicted APDs were then compared. The experiment has been repeated $N = 100$ times. Results are in Fig. 2, where we report the average and the 2σ confidence interval. As it can be seen, the restitution curve based on S1-S2 protocol is more accurate.

Finally, we remark that the S1-S2 protocol does not impede the simulation of alternans. To test this, we paced the Courtemanche model at very high frequency in 0-D, with a BCL of 95 ms. Alternans with APD respectively of 80 ms and 58 ms resulted from the simulation. By iterating the S1-S2 restitution, for the same BCL we obtained a 2-cycle with APD of 78.5 ms and 62 ms, not too far from those observed numerically.

4. Conduction velocity (CV) restitution curves were computed for a given mesh size and tissue conductivity. However, for a specific tissue conductivity, the numerical CV restitution curve is highly sensitive to mesh size. I believe the authors computed the CV restitution curve using a very fine mesh (0.05 mm, which is quite fine) to ensure it approximates the true conduction velocity restitution for the given tissue conductivity. Please make this explicit in the text.

Thanks for acknowledging this. We added the following sentence to Sec.2.2:

> *The mesh size is sufficiently fine to minimize the error in CV [11].*

5. All the examples presented only show cases of single-rotor re-entries. However, clinically relevant problems, such as atrial fibrillation, are not demonstrated using the present methodology. The authors must demonstrate the capability of the methodology to reproduce multi-front re-entries to illustrate its true potential for clinical applications.

We believe that the field of application for our methodology is macro-reentry rather than complex atrial fibrillation events. With our method (and several similar methods recently published by other groups) it would be very hard to correctly capture the meandering of the tip and the interaction between multiple waves. On the other hand, the applicability of our method remains relevant, since macro-reentries such as tachycardia are very common and potentially of interest for targeted ablation. Atrial fibrillation is only mentioned in the introduction, and to avoid potential confusion we replaced the keyword *atrial fibrillation* with *tachycardia*.

6. Another major concern is the handling of tissue anisotropy. To address anisotropy, the algorithm must work with anisotropic meshes. The examples shown in the manuscript are all 2D, i.e., rectangular patches and shell models of the ventricle and atria, with simplified and unrealistic fiber orientations (e.g., circumferential for the ventricle and projections of the $(1, 0, 0)$ unit vector onto the atrial surface). In reality, fiber orientation varies by position—transmurally in the ventricle and regionally in the heart. Generating anisotropic meshes in such cases is challenging, particularly for three-dimensional problems, as well as in the atria, where fiber orientation changes from

region to region. This poses a significant limitation to the method.

Thanks for raising this point. In the revised version we switched to an open database [5] for the atrial experiment (LA for patient 1), which has a more realistic fiber orientation. The mesh adaptivity procedure works as expected. Using an anisotropic ratio of 0.4 between transverse and longitudinal velocities, the number of triangles satisfying the causality condition in the original mesh was 26 %. After adapting the mesh, the value increased to 92 %. The mesh generation took in total 24.42 s on a laptop computer. Please note that we consider this case particularly challenging: first, we needed to extrapolate the fibers from the original mesh to the adapted one. In principle, this step can be improved with rule-based fiber generation algorithms. Second, in the atria the fiber direction can abruptly change around veins and the mitral ring. Most of the invalid triangles are in fact located around regions with discontinuous fiber directions.

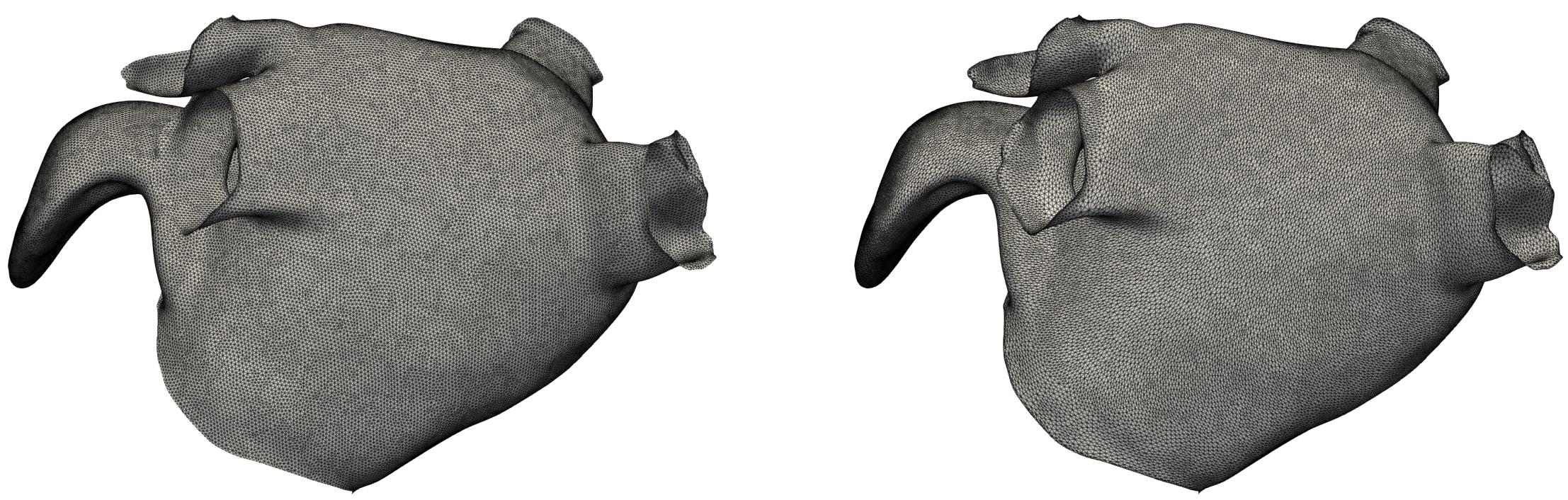

Figure 3: Atrial anatomy from [5] before (left) and after (right) mesh adaptation.

We also remark that the adaptivity can be done once the anisotropy ratio is fixed: the actual value of the conduction velocities and restitution properties do not impact the computation.

Minor Points:

1. In the abstract, the authors state: "... the monodomain model is computationally costly and has limited applicability in practice." While it is true that the monodomain model is a simplification of the bidomain model, it remains widely used by the modeling community to describe cardiac electrophysiology, as it accommodates detailed electrophysiological models of cardiac cells. Considering that the Eikonal model only provides activation times and does not incorporate detailed cellular electrophysiology (or includes information limited to restitution behavior), it is even more limited than the monodomain model. Please reword this statement or consider removing it.

Thanks for noticing it, we rephrased it as follows:

> *However, detailed models, such as the monodomain model, are computationally costly and therefore have limited applicability in the clinical practice.*

2. In the third line of the abstract: "Thus, it desirable . . . " should be corrected to "Thus, it is desirable . . . ".

Fixed.

3. In Algorithm 1, the terms $N(X_0)$ and $\mathrm{HL}(\cdot)$ are not explained. I believe HL stands for the Hopf-Lax formula. Please clarify this in the manuscript.

Fixed.

4. In Algorithm 2, the terms $a(\cdot)$ and $c(\cdot)$ are not defined. The nomenclature must be clearly described in the text for better understanding.

Thanks for noticing. We added the following:

> *The algorithm makes use of the restitution curves $c(\mathrm{DI})$ and $a(\mathrm{DI})$, respectively for the conduction velocity and the APD, at a given diastolic interval DI. The curves are linearly interpolated.*

5. For completeness, it would be helpful to include a figure—at least in the appendix—showing the actual fiber direction and the mesh used in the computations.

Fixed, we added a panel to Figure 8.

References

[1] F. Maffessanti, T. Jadczyk, R. Kurzelowski, F. Regoli, M. L. Caputo, G. Conte, K. S. Gołba, J. Biernat, J. Wilczek, M. Dabrowska, S. Pezzuto, T. Moccetti, R. Krause, W. Wojakowski, F. W. Prinzen, and A. Auricchio. "The influence of scar on the spatio-temporal relationship between electrical and mechanical activation in heart failure patients". In: *EP Europace* 22.5 (2020), pp. 777–786.

[2] S. Pezzuto, F. W. Prinzen, M. Potse, F. Maffessanti, F. Regoli, M. L. Caputo, G. Conte, R. Krause, and A. Auricchio. "Reconstruction of three-dimensional biventricular activation based on the 12-lead electrocardiogram via patient-specific modelling". In: *EP Europace* 23.4 (2021), pp. 640–647.

[3] M. Potse, D. Krause, W. Kroon, R. Murzilli, S. Muzzarelli, F. Regoli, E. Caiani, F. W. Prinzen, R. Krause, and A. Auricchio. "Patient-specific modelling of cardiac electrophysiology in heart-failure patients". In: *Europace* 16.suppl_4 (2014), pp. iv56–iv61.

[4] A. Gharaviri, E. Bidar, M. Potse, S. Zeemering, S. Verheule, S. Pezzuto, R. Krause, J. G. Maessen, A. Auricchio, and U. Schotten. "Epicardial fibrosis explains increased endo-epicardial dissociation and epicardial breakthroughs in human atrial fibrillation". In: *Frontiers in Physiology* 11.68 (2020).

[5] P. Martínez Díaz, J. Sánchez Arciniegas, N. Fitzen, U. Ravens, O. Dössel, and A. Loewe. *The Right Atrium Affects In Silico Arrhythmia Vulnerability in Both Atria*. Version 1.0. Zenodo, Feb. 2024.

[6] C. Dobrzynski and P. Frey. "Anisotropic Delaunay mesh adaptation for unsteady simulations". In: *Proceedings of the 17th International Meshing Roundtable*. Springer, 2008, pp. 177–194.

[7] C. Roney, G. Plank, S. Honarbakhsh, C. Vidal Horrach, S. Pezzuto, and E. Vigmond. "Recovering intrinsic conduction velocity and action potential duration from electroanatomic mapping data using curvature". In: *Medical Image Analysis* 107 (A Jan. 2026), p. 103809.

[8] L. Gander, R. Krause, M. Weiser, F. Sahli Costabal, and S. Pezzuto. "On the accuracy of eikonal approximations in cardiac electrophysiology in the presence of fibrosis". In: *International Conference on Functional Imaging and Modeling of the Heart*. 2023, pp. 137–146.

[9] M. Courtemanche, J. P. Keener, and L. Glass. "A delay equation representation of pulse circulation on a ring in excitable media". In: *SIAM Journal on Applied Mathematics* 56.1 (1996), pp. 119–142.

[10] M. Courtemanche, R. J. Ramirez, and S. Nattel. "Ionic mechanisms underlying human atrial action potential properties: insights from a mathematical model". In: *American Journal of Physiology - Heart and Circulatory Physiology* 275.1 (1998), H301–H321.

[11] S. Pezzuto, J. Hake, and J. Sundnes. "Space-discretization error analysis and stabilization schemes for conduction velocity in cardiac electrophysiology". In: *International Journal for Numerical Methods in Biomedical Engineering* 32.10 (2016), e02762.

Re: JP-RP-2025-287281R1 "An eikonal model with re-excitability for fast simulations in cardiac electrophysiology" by Lia Gander, Rolf Krause, Francisco Sahli Costabal, and Simone Pezzuto

Dear Dr Pezzuto,

Thank you for submitting your manuscript to The Journal of Physiology. It has been assessed by a Reviewing Editor and by 2 expert referees and we are pleased to tell you that it is potentially acceptable for publication following satisfactory major revision.

Please advise your co-authors of this decision as soon as possible.

The referee reports are copied at the end of this email.

Please address all the points raised and incorporate all requested revisions or explain in your Response to Referees why a change has not been made. We hope you will find the comments helpful and that you will be able to return your revised manuscript within 2 months. If you require an extension, please contact journal staff: jp@physoc.org. Please note that this letter does not constitute a guarantee for acceptance of your revised manuscript.

Your revised manuscript should be submitted online using the link in your Author Tasks: Link Not Available. This link is accessible via your account as Corresponding Author; it is not available to your co-authors. If this presents a problem, please contact journal staff (jp@physoc.org). Image files from the previous version are retained on the system. Please ensure you replace or remove any files that are being revised.

If you do not wish to submit a revised version of your manuscript, you must inform our journal staff (jp@physoc.org) or reply to this email to request withdrawal. Please note that a manuscript must be formally withdrawn from the peer review process at one journal before it may be submitted to another journal.

TRANSPARENT PEER REVIEW POLICY: To improve the transparency of its peer review process, The Journal of Physiology publishes online as supporting information the peer review history of all articles accepted for publication. Readers will have access to decision letters, including Editors' comments and referee reports, for each version of the manuscript, as well as any author responses to peer review comments. Referees can decide whether or not they wish to be named on the peer review history document.

ABSTRACT FIGURES: Authors are expected to use The Journal's premium BioRender account to create/redraw their Abstract Figures. Information on how to access this account is here: https://physoc.onlinelibrary.wiley.com/journal/14697793/biorender-access.

This will enable Authors to create and download high-resolution figures. If authors have used the free BioRender service, they can use the instructions provided in the link above to download a high-resolution version suitable for publication.

The link provided should only be used for the purposes of this submission. Authors will be charged for figures created on this account if they are not related to this manuscript submission.

LANGUAGE EDITING AND SUPPORT FOR PUBLICATION: If you would like help with English language editing, or other article preparation support, Wiley Editing Services offers expert help, including English Language Editing, as well as translation, manuscript formatting, and figure formatting at www.wileyauthors.com/eeo/preparation. You can also find resources for Preparing Your Article for general guidance about writing and preparing your manuscript at www.wileyauthors.com/eeo/prepresources.

RIGOUR AND REPRODUCIBILITY: We recommend authors consult The Journal's Rigour and Reproducibility requirements to ensure that their manuscript is suitable for publication: https://physoc.onlinelibrary.wiley.com/pb-assets/hub-assets/physoc/documents/TJP-Rigour-and-Reproducibility-Requirements.pdf

REVISION CHECKLIST:

Check that your Methods section conforms to journal policy: https://jp.msubmit.net/cgi-bin/main.plex?form_type=display_requirements#methods.

Check that data presented conforms to the statistics policy: https://jp.msubmit.net/cgi-bin/main.plex?form_type=display_requirements#statistics.

Upload a full Response to Referees file. To create your 'Response to Referees': copy all the reports, including any comments from the Senior and Reviewing Editors, into a Microsoft Word, or similar, file and respond to each point, using font or background colour to distinguish comments and responses and upload as the required file type.

Please upload two versions of your manuscript text: one with all relevant changes highlighted and one clean version with no

changes tracked. The manuscript file should include all tables and figure legends, but each figure/graph should be uploaded as separate, high-resolution files.

You may also upload:

- 'Potential Cover Art' for consideration as the issue's cover image
- Appropriate Supporting Information (Video, audio or data set: see https://jp.msubmit.net/cgi-bin/main.plex?form_type=display_requirements#supp).

We look forward to receiving your revised submission.

If you have any queries, please reply to this email and we will be pleased to advise.

Yours sincerely,

Natalia Trayanova
Senior Editor
The Journal of Physiology

REQUIRED ITEMS

(1) Please include a Key Points list in the article itself, before the Abstract.

(2) Please include an Abstract Figure file and an Abstract Figure legend (** we seem to be missing the legend **). An appropriate figure legend, which should not exceed 150 words in length, should be included in the main manuscript file. The Abstract Figure is a piece of artwork designed to give readers an immediate understanding of the research and should summarise the main conclusions. If possible, the image should be easily 'readable' from left to right or top to bottom. It should show the physiological relevance of the manuscript so readers can assess the importance and content of its findings. Abstract Figures should not merely recapitulate other figures in the manuscript. Please try to keep the diagram as simple as possible and without superfluous information that may distract from the main conclusion(s). Abstract Figures must be provided by authors no later than the revised manuscript stage and should be uploaded as a separate file during online submission labelled as File Type 'Abstract Figure'. Please also ensure that you include the figure legend in the main article file. All Abstract Figures should be created using BioRender. Authors should use The Journal's premium BioRender account to export high-resolution images. Details on how to use and access the premium account are included as part of this email.

EDITOR COMMENTS

Reviewing Editor:

The Referees completed their review of the revised version of the paper. Referee 2 would like to have more details on point 6 (see below), so please provide a detailed and satisfactory answer:

REF2: The authors have address most of my concerns. With regard point 6 of the previous review:

6) Another major concern is the handling of tissue anisotropy. To address anisotropy, the algorithm must work with anisotropic meshes. The examples shown in the manuscript are all 2D, i.e., rectangular patches and shell models of the ventricle and atria, with simplified and unrealistic fiber orientations (e.g., circumferential for the ventricle and projections of the (1, 0, 0) unit vector onto the atrial surface). In reality, fiber orientation varies by position-transmurally in the ventricle and regionally in the heart. Generating anisotropic meshes in such cases is challenging, particularly for three-dimensional problems, as well as in the atria, where fiber orientation changes from region to region. This poses a significant limitation to the method.

The authors have not responded to the actual comment. They have changed the atrial model, but it is still 2D. The comment referred to the case in which the model is 3D (with actual thickness). In this case, the fibre orientation also changes transmurally, making remeshing more difficult. The reviewer sees this as a limitation that needs to be addressed in the discussion.

Please also see 'Required Items' above.

Senior Editor:

The authors need to include physiological insight in the manuscript. Without new physiological insight, the paper does not belong to the Journal of Physiology, Also, there is a remaining issue to be addressed in response to the comment by Reviewer 2.

If the authors can make such major changes to the manuscript, and provide new mechanistic insight, we would be happy to re-evaluate. However, the paper simply is not written for this journal's audience, and the authors might find a better suiting journal for their research.

REFEREE COMMENTS

Referee #1:

The authors have responded to all of my concerns except for the boundary condition of the Eikonal equation. They have neglected the zero normal flux condition which is not considered with marching cubes. The effect is minor.

Referee #2:

The authors have address most of my concerns. With regard point 6 of the previous review:

6) Another major concern is the handling of tissue anisotropy. To address anisotropy, the algorithm must work with anisotropic meshes. The examples shown in the manuscript are all 2D, i.e., rectangular patches and shell models of the ventricle and atria, with simplified and unrealistic fiber orientations (e.g., circumferential for the ventricle and projections of the (1, 0, 0) unit vector onto the atrial surface). In reality, fiber orientation varies by position-transmurally in the ventricle and regionally in the heart. Generating anisotropic meshes in such cases is challenging, particularly for three-dimensional problems, as well as in the atria, where fiber orientation changes from region to region. This poses a significant limitation to the method.

The authors have not responded to the actual comment. They have changed the atrial model, but it is still 2D. The comment referred to the case in which the model is 3D (with actual thickness). In this case, the fibre orientation also changes transmurally, making remeshing more difficult. The reviewer sees this as a limitation that needs to be addressed in the discussion.

END OF COMMENTS

An eikonal model with re-excitability for fast simulations in cardiac electrophysiology

Lia Gander, Rolf Krause, Francisco Sahli Costabal, Simone Pezzuto

Reviewing Editor

The Referees completed their review of the revised version of the paper. Referee 2 would like to have more details on point 6 (see below), so please provide a detailed and satisfactory answer.

We thank the Reviewing Editor for the opportunity to clarify this remaining point. We have revised the Discussion to explicitly address the limitation related to full three-dimensional anisotropic remeshing with transmural fibre rotation, as requested by Rev. #2.

Please also see 'Required Items':

(1) Please include a Key Points list in the article itself, before the Abstract.

*(2) Please include an Abstract Figure file and an Abstract Figure legend (** we seem to be missing the legend **). An appropriate figure legend, which should not exceed 150 words in length, should be included in the main manuscript file.*

Fixed, thanks for noting.

Senior Editor - Comments

The authors need to include physiological insight in the manuscript. Without new physiological insight, the paper does not belong to the Journal of Physiology, Also, there is a remaining issue to be addressed in response to the comment by Reviewer 2.

If the authors can make such major changes to the manuscript, and provide new mechanistic insight, we would be happy to re-evaluate. However, the paper simply is not written for this journal's audience, and the authors might find a better suiting journal for their research.

We thank the Senior Editor for this frank assessment. We understand the concern that the manuscript may have appeared too strongly focused on numerical methodology. We have therefore made targeted revisions to better articulate the physiological insight provided by the model, without changing the scope of the work or overstating its conclusions.

The central physiological point is that the proposed formulation separates two mechanisms that jointly determine macro-reentry. The first is the anatomical and conductive substrate that determines where a wave can propagate, including fibre orientation, conduction velocity, scar and non-conductive barriers. The second is the local recovery of the tissue, described through restitution of action potential duration and conduction velocity. This separation provides a controlled way to study how changes in local tissue physiology, including electrophysiological remodelling or drug-induced changes in refractoriness and conduction, alter the vulnerability of a given anatomical substrate to re-entry.

We added this point explicitly to the Abstract, Introduction and Discussion. In particular, the Discussion now states:

> *The resulting model is not intended to replace detailed biophysical simulations, but to provide a controlled way to separate the substrate for wave propagation from the restitution properties that determine when tissue can be re-excited. In the examples considered here, re-entry is sustained only when the anatomical pathway, conduction anisotropy and local recovery time are mutually compatible. This provides a mechanistic interpretation of the simulations: changes in conduction pathway or tissue recovery can be varied independently to assess whether they promote unidirectional block, re-excitation and maintenance of macro-reentry.*

We further expanded the Discussion to clarify why this mechanistic separation is important in the broader context of cardiac digital twins. Clinical applications such as inducibility testing, pacing-site screening, parameter exploration and virtual ablation require many repeated simulations, so speed is not only a computational convenience but a practical requirement. We now distinguish the proposed approach from purely data-driven emulators and neural-network surrogates: such methods can be very fast at inference, but their applicability depends on the high-fidelity simulations used for training and on the variables selected for parametrization, including anatomy, scar or fibrosis distribution, conduction properties, pacing protocol and outputs of interest. In contrast, our model does not learn a patient-specific input-output map. It

keeps propagation and recovery as explicit physiological components, making it modular and interpretable while retaining the potential for near-real-time performance and preserving qualitative agreement with monodomain simulations in the macro-reentry cases considered.

In particular, we added:

> *This structure makes the model transparent and modular: specific changes in tissue recovery or conduction can be tested for their effect on unidirectional block, re-excitation and maintenance of macro-reentry, while retaining the potential for near-real-time performance and preserving qualitative agreement with monodomain simulations in the clinically relevant macro-reentry cases considered here.*

We also added a Key Points summary and an Abstract Figure legend to present the physiological message more clearly for the journal's audience. In addition, we clarified in the restitution subsection why the S1-S2 protocol is appropriate for the acute tissue response relevant to re-entry, whereas dynamic restitution protocols probe longer-term adaptation under sustained pacing.

Finally, we respectfully note that this manuscript was commissioned as part of the proceedings of the INdAM workshop *Mathematical and Numerical Modeling of the Cardiovascular System*, held in Rome on 15–19 April 2024. Accordingly, the work naturally contains a numerical-methods component. We believe this is consistent with the journal's *Computational Physiology and Modelling* section and with other papers from the same conference that have appeared in *The Journal of Physiology* with substantial computational and numerical content, for example papers on atrial fibre inference (JP288001), computational ECG modelling in left ventricular hypertrophy (JP287954), ventricular repolarization and arrhythmia vulnerability (JP288356), in silico modelling for cardiac drug testing (JP287276), and lumped heart models for personalized medicine (JP287929). We have nevertheless revised the manuscript to make the physiological motivation and mechanistic interpretation more explicit.

Reviewer #2 - Comments

The authors have address most of my concerns. With regard point 6 of the previous review:

6. Another major concern is the handling of tissue anisotropy. To address anisotropy, the algorithm must work with anisotropic meshes. The examples shown in the manuscript are all 2D, i.e., rectangular patches and shell models of the ventricle and atria, with simplified and unrealistic fiber orientations (e.g., circumferential for the ventricle and projections of the (1, 0, 0) unit vector onto the atrial surface). In reality, fiber orientation varies by position-transmurally in the ventricle and regionally in the heart. Generating anisotropic meshes in such cases is challenging, particularly for three-dimensional problems, as well as in the atria, where fiber orientation changes from region to region. This poses a significant limitation to the method.

The authors have not responded to the actual comment. They have changed the atrial model, but it is still 2D. The comment referred to the case in which the model is 3D (with actual thickness). In this case, the fibre orientation also changes transmurally, making remeshing more difficult. The reviewer sees this as a limitation that needs to be addressed in the discussion.

We thank the reviewer for clarifying the point. We agree that our previous response did not address the specific issue of full three-dimensional myocardium with transmural fibre rotation. The examples in the manuscript are surface models, and therefore they do not test the more difficult case of a full-thickness ventricular mesh with a fibre field varying through the wall.

We have now explicitly added this limitation to the Discussion. We also clarified that three-dimensional anisotropic mesh generation is possible in principle, as shown for example in the work of Rouxel-Labbé [1], but that the specific requirement of our FMM formulation is more restrictive: the tetrahedral mesh must satisfy the metric-acuteness constraints associated with the anisotropic conduction tensor. In a full-thickness cardiac model, this tensor would vary transmurally with the fibre orientation, and satisfying these constraints may require substantially finer tetrahedral meshes, reducing the practical speed-up.

We added the following text to the Discussion:

> *Importantly, the present surface formulation does not resolve transmural fiber rotation in full-thickness myocardium. General three-dimensional anisotropic mesh-generation strategies have been developed and tested in a variety of metric fields [1], and therefore a full-thickness extension is not precluded in principle. In a ventricular model, however, anisotropic remeshing would have to accommodate a fiber field that varies through*

the wall while preserving the metric-acuteness constraints required by the FMM. This may require substantially finer tetrahedral meshes and could reduce the practical speed-up. Extension to full-thickness anatomies with transmural fiber rotation therefore remains an important limitation of the present work and a target for future developments.

References

[1] M. Rouxel-Labbé. “Génération de maillages anisotropes”. PhD thesis. Université Côte d’Azur, 2016.

Dear Dr Pezzuto,

Re: JP-RP-2026-287281R2 "An eikonal model with re-excitability for fast simulations in cardiac electrophysiology" by Lia Gander, Rolf Krause, Francisco Sahli Costabal, and Simone Pezzuto

We are pleased to tell you that your paper has been accepted for publication in The Journal of Physiology.

The last Word (or similar) version of the manuscript provided will be used by the Production Editor to prepare your proof. When this is ready you will receive an email containing a link to Wiley's Online Proofing System. The proof should be thoroughly checked and corrected as promptly as possible.

Authors should note that it is too late at this point to offer corrections prior to proofreading. Major corrections at proof stage, such as changes to figures, will be referred to the Editors for approval before they can be incorporated. Only minor changes, such as to style and consistency, should be made at proof stage. Changes that need to be made after proof stage will usually require a formal correction notice.

All queries at proof stage should be sent to: TJP@wiley.com.

Following copyediting and typesetting the accepted version will be published online.

Yours sincerely,

Natalia Trayanova
Senior Editor
The Journal of Physiology

IMPORTANT POINTS TO NOTE FOLLOWING ACCEPTANCE OF YOUR PAPER:

• IMPORTANT NOTICE ABOUT OPEN ACCESS: To assist authors whose funding agencies mandate immediate public access to published research findings, The Journal of Physiology allows authors to pay an Open Access (OA) fee to have their papers made freely available immediately on publication.

The Corresponding Author will receive an email from Wiley with details on how to register or log in to Wiley Authors where you will be able to place an order.

• You can check if your funder or institution has a Wiley Open Access Account here:
https://authors.wiley.com/author-resources/Journal-Authors/open-access/author-compliance-tool.html

MEMBER DISCOUNT: Corresponding authors who are Members of the Physiological Society are eligible for a 20% discount on the cost of the Article Processing Charge (APC). To claim this discount, the Corresponding Author will need to log in to the Physiological Society Membership portal and retrieve the discount code (https://www.physoc.org/journals-and-media/journals/journal-discount-for-members/). This code should then be quoted in the billing section of Wiley Authors when selecting the open access option.

• TRANSPARENT PEER REVIEW POLICY: To improve the transparency of its peer review process, The Journal of Physiology publishes online as supporting information the peer review history of all articles accepted for publication. Readers will have access to decision letters, including Editors' comments and referee reports, for each version of the manuscript as well as any author responses to peer review comments. Referees can decide whether or not they wish to be named on the peer review history document.

• You can help your research get the attention it deserves! Check out Wiley's free Promotion Guide for best-practice recommendations for promoting your work at: www.wileyauthors.com/eeo/guide. You can learn more about Wiley Editing Services which offers professional video, design, and writing services to create shareable video abstracts, infographics, conference posters, lay summaries, and research news stories for your research at: www.wileyauthors.com/eeo/promotion.

• If you would like to receive our 'Research Roundup', a monthly newsletter highlighting the cutting-edge research published in The Physiological Society's family of journals (The Journal of Physiology, Experimental Physiology, Physiological Reports, The Journal of Nutritional Physiology and The Journal of Precision Medicine: Health and Disease), please click this link, fill in your name and email address and select 'Research Roundup':
https://www.physoc.org/journals-and-media/membernews

EDITOR COMMENTS

Reviewing Editor:

The Referees are both happy with the final answers and version of the paper.

REFEREE COMMENTS

Referee #1:

The authors have adequately responded to the questions.

Referee #2:

The authors have addressed the comment by including in the discussion the limitations of their model to properly handle tissue anisotropy in 3D tissue architecture.
